# Low-Density sEMG Four-Finger Recalibration with Limited Calibration Data via Classwise Amplitude and Covariance Adaptation of Prior Training Data

**DOI:** 10.3390/s26216921

**Published:** 2026-10-31

**Authors:** Jiwoong Won, Masami Iwase

**Affiliations:** Advanced Multidisciplinary Engineering, Graduate School of Advanced Science and Technology, Tokyo Denki University, Tokyo 120-8551, Japan; iwase@ctrl.fr.dendai.ac.jp

**Keywords:** surface electromyography (sEMG), low-density sEMG, finger-motion classification, sensor re-donning, personalized recalibration, training-data adaptation, covariance alignment

## Abstract

**Highlights:**

**What are the main findings?**

- In this three-participant feasibility study, when participant-specific Training data were adapted to the current wearing condition and reused after sensor re-donning, classwise amplitude correction and classwise covariance alignment recovered four-finger Stable Motion accuracy from 88.90% to 98.88% for P1 across all ten Test datasets from five re-donning conditions. For P2 and P3, an exploratory post hoc analysis examined the four cross-condition directions in which the fixed classifier showed substantial degradation. Within these failure-case subsets, accuracy increased from 24.46% to 98.97% and from 73.74% to 96.72%, respectively.
- In exhaustive retrospective shared-subset analyses, fold-nested two-repetition combined accuracy remained within 0.18–0.58 percentage points of the corresponding full-five accuracy for P1–P3.

**What is the implication of the main finding?**

- These findings provide preliminary feasibility evidence that previously acquired participant-specific Training data can be adapted and reused after sensor re-donning, potentially reducing the amount of newly acquired Calibration data required for classifier reconstruction. In the retrospective repetition-subset analysis, reducing Calibration from five to two repetitions per finger retained high classification accuracy and corresponded to a theoretical protocol-duration reduction from approximately 159 s to 87 s (45%). This duration reduction is a practical implication calculated from the protocol timing, not a prospectively validated acquisition result.

**Abstract:**

Sensor re-donning can alter surface electromyography (sEMG) amplitudes and inter-channel relationships, degrading sEMG-based motion classifiers. This study proposes personalized recalibration for four-finger motion classifiers and evaluates whether reusing prior Training data can reduce newly acquired Calibration data. Limited labeled Calibration data transform Training signals toward the current condition via classwise amplitude correction and classwise regularized covariance alignment before support vector machine retraining. Three able-bodied male participants (P1–P3) were evaluated using Stable Motion window-level accuracy. For P1, accuracy pooled over ten Test datasets from five re-donning conditions increased from 88.90% to 98.88%. For P2 and P3, exploratory post hoc analyses of four failure-case directions per participant showed pooled accuracy increasing from 24.46% to 98.97% and from 73.74% to 96.72%, respectively. Across exhaustive retrospective analyses of all shared two-of-five repetition subsets, Combined accuracy was 98.29%, 98.68%, and 95.58% for P1–P3, respectively, only 0.18–0.58 percentage points below the five-repetition results; prospectively shortened Calibration recordings were not evaluated. These findings support the feasibility of participant-specific recalibration for Stable Motion four-finger classification after sensor re-donning and warrant further evaluation in broader user populations and continuous-operation settings.

## 1. Introduction

Surface electromyography (sEMG) provides information related to muscle activation and has been widely used as a control signal for human–machine interfaces (HMIs). In particular, sEMG-based motion recognition has been extensively investigated for myoelectric prosthetic systems because it enables user-intended hand motions to be reflected in device control. For practical and continuous use, however, an sEMG-based HMI must not only achieve high performance under a specific measurement condition but also maintain reliable performance across repeated use and measurement sessions.

A fundamental challenge in sEMG-based interfaces is the substantial variability of sEMG signals across individuals, which makes it difficult to directly apply a model trained on one user or group of users to a new user. In particular, pattern-recognition-based myoelectric interfaces commonly acquire sEMG data from the end user through a calibration or training procedure and use these data to construct a user-specific classification model [1,2]. Such personalization reduces the influence of inter-user variability by constructing a classifier that reflects the muscle activation patterns of the individual user.

However, even a classifier personalized using user-specific data does not necessarily maintain the same performance across repeated measurement sessions. The observed sEMG characteristics can vary when the measurement condition changes, even when the same user performs the same motion, resulting in intra-user cross-session variability. In particular, repeated sensor donning and doffing can change electrode contact locations relative to those in the original measurement session. Young et al. reported that electrode displacement can degrade myoelectric pattern-recognition performance and that the magnitude of this effect depends on the displacement condition [3]. Thus, sensor re-donning does not necessarily make an existing model unusable in every case; however, classification performance may deteriorate when the current measurement condition differs sufficiently from the condition represented in the training data.

In a multi-channel sEMG system, electrode displacement can affect not only the signal magnitude of individual channels but also the relative signal characteristics across channels. Tanaka et al. showed that sliding-window normalization of sEMG amplitudes can mitigate the reduction in classification accuracy caused by electrode displacement [4]. This finding indicates that changes in relative channel-amplitude characteristics are relevant to classification robustness across sensor-placement conditions.

Yan et al. more directly addressed electrode re-wearing and radial position shifts using adaptive myo-spatial correction [5]. Their method estimated an electrode-shift angle from an EIT-like sEMG feature map and rotated the current feature map toward the original feature space. This provides a recent example of explicitly position-informed correction. In contrast, the present study used labeled classwise statistics from the current wearing condition to adapt prior participant-specific raw Training signals toward naturally occurring re-donning conditions without requiring explicit physical-displacement estimation.

Moreover, multi-channel sEMG contains spatial information represented by the relationships among signals recorded at different electrode locations, in addition to the signal characteristics of individual channels. Stango et al. demonstrated that spatial correlation features derived from multi-channel sEMG can provide robustness against electrode shift [6]. More recently, Gowda and Miller constructed covariance matrices from pairwise relationships among sEMG signals recorded at different electrodes to represent the spatial patterns of multi-channel muscle activity. Such covariance-based representations can characterize sEMG variability associated with gestures, individuals, and recording sessions [7].

Consequently, changes in the measurement condition caused by sensor re-donning can alter both the channel-wise amplitude characteristics and the inter-channel spatial relationships of multi-channel sEMG, resulting in a distribution mismatch between previously acquired user-specific training data and data observed in the current measurement session. When this mismatch becomes sufficiently large, the decision boundary of a personalized classifier trained under the previous measurement condition may no longer be appropriate for the current data distribution, leading to degraded classification performance. Conversely, when the current measurement condition remains sufficiently similar to the original training condition, the existing model may remain effective. This suggests that the need for recalibration may depend on the extent to which the current measurement condition differs from the original training condition.

One approach to improving cross-session robustness is to incorporate sEMG data acquired from multiple measurement sessions into the training dataset. Previous studies have shown that increasing the number of training sessions can be more effective for improving cross-session classification performance than simply increasing the number of repetitions within a single session [8]. This suggests that incorporating signal variability across different measurement sessions into the training process can improve the robustness of a classifier to subsequent sessions. However, constructing such a model requires repeated acquisition of user-specific training data under multiple measurement conditions, which increases the time and effort required from the user [1]. Moreover, there are practical limitations to anticipating the various measurement conditions that may occur during long-term use and acquiring sufficient training data for all of them in advance. When a substantial mismatch nevertheless occurs after sensor re-donning, the most direct response is to collect a sufficiently large labeled dataset under the current wearing condition and train a new classifier. Repeating this procedure after each re-donning would again require the user to perform many labeled finger-motion trials. Therefore, a practical recalibration strategy should limit the amount of newly acquired data while effectively reusing previously acquired user-specific training information. This raises the question of whether previously acquired user-specific training data can be adapted to a current wearing condition using only limited labeled calibration data while recovering classification performance after sensor re-donning.

Previous studies have investigated recalibration and adaptation methods that use a limited amount of data acquired from a new measurement session to recover classification performance. Vidovic et al. proposed a supervised covariate-shift adaptation method in which a previously trained classifier is adapted to the signal distribution of a new session using less than one minute of labeled calibration data, thereby mitigating performance degradation across measurement days [9]. Zhu et al. proposed a cascaded adaptation framework that automatically incorporates models learned from previous sessions into the calibration of the current session and updates model parameters as new data become available [10].

Other studies have focused on transforming or selectively acquiring current-session data to reduce the amount of data required for adaptation. Karrenbach et al. proposed a session-specific recalibration architecture in which a calibrator trained using a small portion of data from a new session transforms current-session inputs into a more session-independent representation while retaining a model learned from multiple previous sessions [11]. Szymaniak et al. investigated an active-learning-based recalibration framework that selectively queries informative samples from current data and iteratively modifies the classifier decision boundaries, thereby reducing the amount of labeled data required for adaptation [12]. Collectively, these studies have addressed cross-session sEMG variability through different strategies, including adapting classifier parameters, incorporating information from previous-session models, transforming current-session inputs, and selectively updating decision boundaries.

Recent studies have further examined transfer learning and domain adaptation for sEMG distribution shifts. Shi et al. systematically compared eight unsupervised transfer-learning methods for multi-user gesture recognition and reported CORAL-based alignment under limited-Calibration, cross-gesture, and cross-day settings [13]. Guo et al. proposed an inter-session domain-adaptation method combining feature-distribution alignment, locality preservation, maximum-margin separation, and iterative pseudo-label selection [14]. These approaches adapt model parameters or extracted feature representations across users or sessions, whereas the present framework uses labeled classwise statistics from the current wearing condition to transform prior participant-specific raw Training signals toward that condition before feature re-extraction and classifier retraining.

These studies demonstrate that cross-session variability can be addressed by combining a previously trained model or information from earlier sessions with a limited amount of current-session data. However, the approaches reviewed above differ in their adaptation targets and processing directions. Some methods use current-session Calibration data to update classifier parameters or decision boundaries, whereas another retains a previously learned model and transforms current-session inputs into a representation suitable for that model. In contrast to the approaches reviewed above, the present study uses class-specific channel-amplitude and covariance characteristics estimated from the Calibration data to transform the Training data themselves toward the current wearing condition and retrains the classifier using the resulting Adapted training data. This direction of adaptation preserves and reuses the information contained in the Training dataset while requiring only a small amount of newly acquired data.

Covariance alignment and its whitening–coloring implementation are established domain-adaptation operations. The present study proposes their classwise application in the raw-signal space as part of a Training-to-current recalibration strategy for sEMG sensor re-donning. In this strategy, labeled classwise statistics from the current wearing condition are used to adapt prior participant-specific Training signals after classwise amplitude correction, followed by feature re-extraction and classifier retraining.

Based on this concept to address the question, this study proposes a personalized recalibration and retraining framework for finger-motion classification using an eight-channel low-density sEMG sensor. To address these two sources of distribution mismatch, classwise amplitude correction and classwise regularized covariance alignment are combined to compensate for changes in signal magnitude and inter-channel structure. The participant-specific data used to construct the initial classifier are hereafter referred to as Training data. A small labeled dataset acquired under the current sensor-wearing condition to estimate its amplitude and covariance characteristics is referred to as Calibration data. The proposed framework transforms the Training data toward the current wearing condition using characteristics estimated from the Calibration data. The transformed data are referred to as Adapted training data; their features are re-extracted and used to retrain the support vector machine (SVM) classifier. Independent Test data are excluded from both training and recalibration and are used only for final performance evaluation.

This study evaluates, in three participants, denoted Participant 1 (P1), Participant 2 (P2), and Participant 3 (P3), the degradation of participant-specific finger-motion classification performance after sensor re-donning and its recovery through the proposed recalibration method. The primary evaluation focuses on Stable Motion windows, in which the sEMG patterns of the Index, Middle, Ring, and Little fingers are sufficiently established for four-finger discrimination. The proposed strategy, which transforms the Training data toward the current wearing condition and retrains the classifier using the resulting Adapted training data, is compared with applying the fixed classifier without adaptation, correcting the current Test signals toward the Training-data condition while retaining the fixed classifier, and constructing a new classifier using only the current Calibration data. To evaluate whether the recalibration burden can be reduced, the amount of Calibration data is varied to determine whether Training-data reuse can maintain high classification performance with only a small amount of Calibration data.

The main contributions of this study are as follows:

- A Training-to-current recalibration direction is proposed for cases in which sensor re-donning degrades a participant-specific classifier. Limited labeled Calibration data characterize the current wearing condition, and classwise amplitude correction and classwise regularized covariance alignment transform the Training data toward that condition. Retraining with the resulting Adapted training data is evaluated as a means of recovering Stable Motion four-finger discrimination.
- An exploratory analysis evaluates the effect of Calibration repetition count by comparing Training-data reuse with Calibration-only retraining. Calibration-only retraining serves as a current-wearing-condition reference: it constructs a classifier directly from data acquired under the same wearing condition as the held-out Test data. The comparison examines whether Training data acquired under a different wearing condition can be transformed toward the current condition and approach this within-wearing reference under the same selected current-Calibration burden. The analysis further examines whether the proposed method can retain high Stable Motion accuracy with fewer Calibration repetitions, thereby indicating the potential to reduce the amount of data required for recalibration and the associated data-acquisition burden.

## 2. Materials and Methods

The overall framework consists of a four-class ECOC-SVM finger-motion classifier and a personalized recalibration procedure. A participant-specific classifier is initially constructed using Training data to distinguish the individual motions of the Index, Middle, Ring, and Little fingers. After sensor re-donning, when recalibration is required, a small amount of labeled Calibration data acquired under the current sensor-wearing condition is used as a reference to transform the Training data toward the current condition, and the classifier is retrained using the resulting Adapted training data. The methodological target is recovery of four-finger discrimination within Stable Motion intervals, in which finger-specific sEMG information is sufficiently established for reliable classification. The three participants are denoted P1, P2, and P3 throughout. The following subsections describe the data acquisition, signal processing, participant-specific classifier, personalized recalibration procedure, the effect of the number of Calibration repetitions, and the evaluation protocols.

### 2.1. Experimental Setup and Data Acquisition

**Figure 1:**
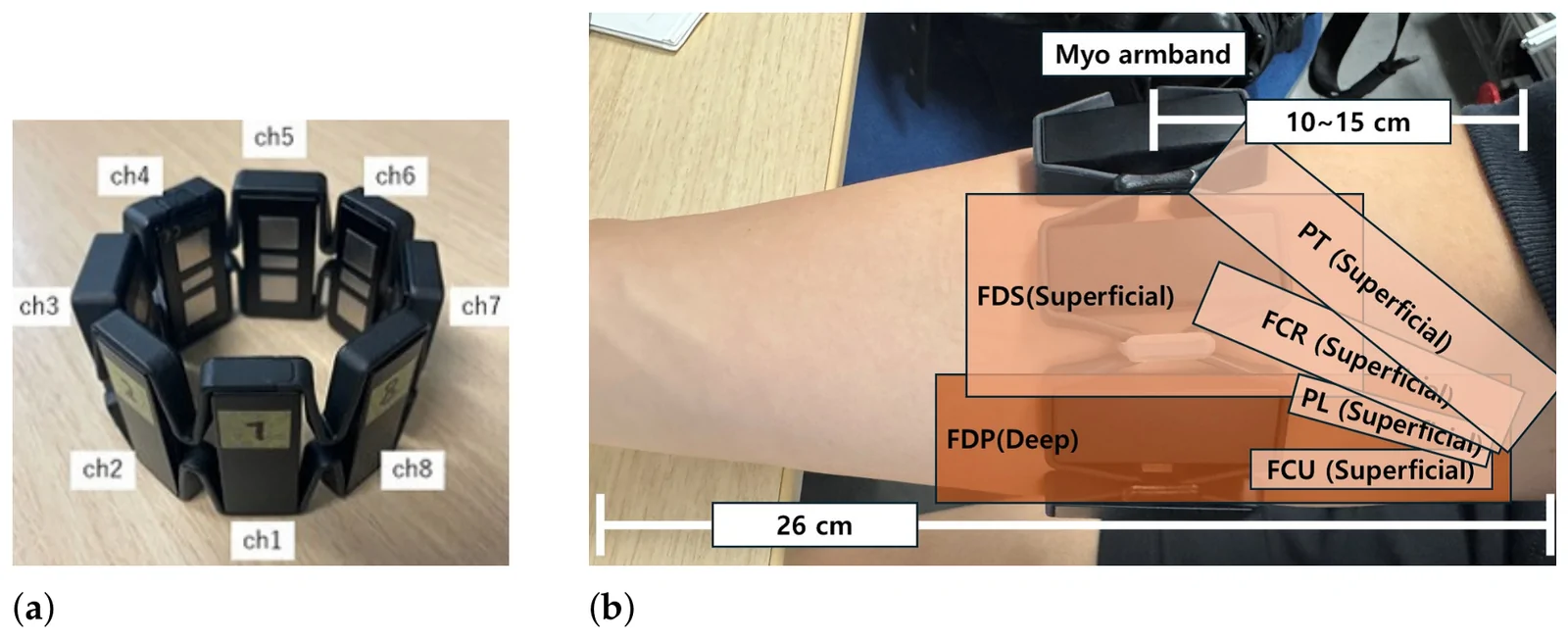
Myo Armband configuration and placement. Panel (**a**) Channel numbering of the eight-channel Myo Armband. It shows the channel numbering defined for analysis. Panel (**b**) Representative placement over the approximate forearm region associated with finger-flexion muscles. It shows a representative participant-specific placement over an approximate forearm region expected to capture sEMG activity from muscles involved in finger flexion. The anatomical overlays indicate the expected muscle regions rather than precise anatomical boundaries. The displayed 26 cm and 10–15 cm dimensions pertain only to the photographed participant and differ for the other participants; they were not used as fixed placement criteria. Abbreviations: FDS, flexor digitorum superficialis; FDP, flexor digitorum profundus; FCR, flexor carpi radialis; FCU, flexor carpi ulnaris; PL, palmaris longus; PT, pronator teres.

sEMG signals were acquired from three able-bodied male participants, denoted Participant 1 (P1), Participant 2 (P2), and Participant 3 (P3), using an eight-channel Myo Armband (Thalmic Labs, Kitchener, ON, Canada) placed around the right forearm. All three participants were in their twenties at the time of data acquisition. For analysis, the eight sensing units were numbered sequentially around the circumference of the armband, as shown in Figure 1a. For each participant, the armband was worn around an approximate forearm region expected to capture sEMG activity from the muscles involved in finger flexion, as illustrated in Figure 1b. The placement was determined separately for each participant and was not defined using a generalized fixed distance. The sEMG signals were acquired by the Myo Armband at 200 Hz and transmitted wirelessly to the acquisition computer via Bluetooth Low Energy. During data acquisition, the participants were seated with the forearm extended forward, the elbow flexed at approximately $90^{\circ }$, and the palm facing downward. The participants performed individual flexion motions of the index, middle, ring, and little fingers. All experimental procedures were conducted in accordance with approved ethical guidelines. Ethical approval was obtained from the Human Life Ethics Committee of Tokyo Denki University (Approval No. 07-010), and written informed consent was obtained prior to the experiment.

Data were recorded using a custom C++ Win32 application with the Thalmic Myo SDK and Myo Connect, and the signed 8-bit EMG samples delivered by the SDK were stored without additional application-level filtering, rectification, normalization, or gain adjustment. The exact SDK, Myo Connect, and device-firmware versions were not preserved. All received packets were saved; BLE gaps were not systematically detected, and no gap reconstruction or interpolation was applied. Offline analysis used the received sample order with the nominal 200-Hz time base. Any internal device-level preprocessing was not characterized. Motion-interval filtering was performed separately during offline analysis.

For each participant, multiple datasets (Dataset 1, Dataset 2, …) were acquired while the sensor remained worn under a given wearing condition. The recordings within the same condition were obtained without removing or repositioning the sensor and therefore represented the same sensor-wearing condition. After the measurements under one condition were completed, the sensor was completely removed and reattached to define the next wearing condition. The experimental grouping was based solely on the sensor-wearing condition: recordings obtained without sensor removal belonged to the same condition, whereas recordings obtained after complete removal and re-donning belonged to a different condition. Acquisition date and elapsed time were not used to define these groups. The sensor was completely removed and subsequently reattached under ordinary wearing conditions. This procedure was used to reproduce measurement-state changes that may naturally occur during repeated sensor donning and doffing rather than artificially introducing a fixed electrode displacement. Accordingly, the experiment was designed to evaluate recalibration under naturally occurring changes in sensor-wearing condition after complete removal and re-donning.

**Figure 2:**
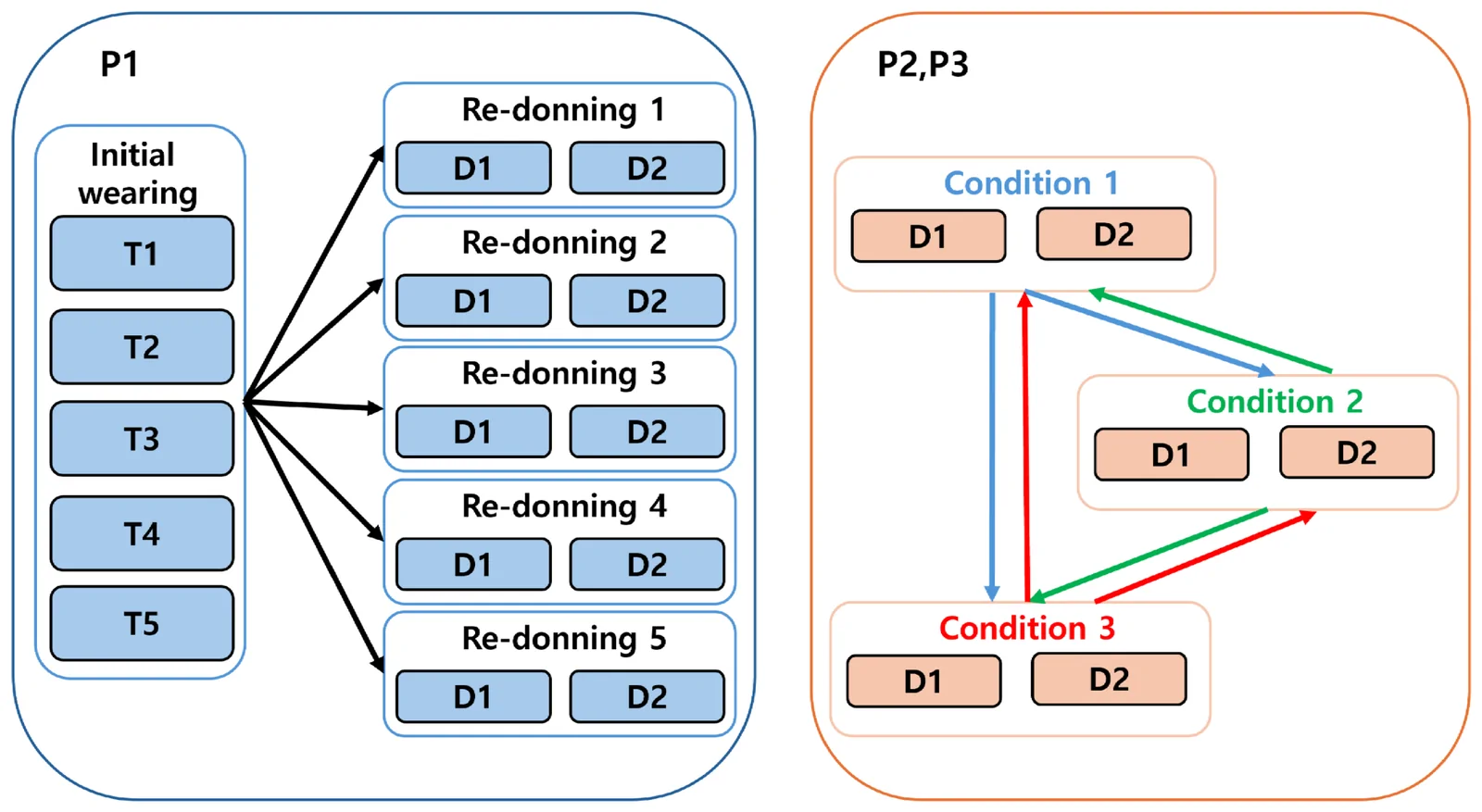
Study design of the two complementary data configurations. Left (P1): five Training datasets (T1–T5) acquired under the initial wearing condition remained the fixed Training source; each of the five subsequent re-donning conditions provided two datasets (D1 and D2) that were assigned to the Calibration and Test roles. Right (P2/P3): three wearing conditions (C1–C3), each with two datasets (D1 and D2), were used in a rotating cross-condition evaluation. Arrow colors identify the corresponding source conditions. The arrows indicate the directed transfers in which both datasets from a source condition were combined for Training and datasets from a target condition were used for Calibration and Test.

The data configurations for P1 and P2/P3 were designed to evaluate complementary experimental objectives and are summarized in Figure 2. For P1, five datasets acquired under the initial wearing condition were used as Training data to construct a stable participant-specific classifier from multiple initial recordings rather than from a single recording. These five Training datasets are denoted T1–T5 throughout. Subsequently, two datasets were acquired under each of five independent sensor re-donning conditions, yielding ten re-donning datasets for evaluation. Within each re-donning condition, the two datasets are denoted D1 and D2 and were assigned to the Calibration and Test roles according to the evaluation direction. This configuration was intended to assess how various sensor re-donning conditions affected the Stable Motion finger-discrimination performance of the existing classifier and how effectively recalibration recovered that performance, while reducing the likelihood that the initial classifier was limited by reliance on only one Training recording.

For P2 and P3, two datasets were acquired consecutively under each of three sensor-wearing conditions, resulting in six datasets per participant. The three wearing conditions were denoted C1, C2, and C3, and the two consecutively acquired datasets within each condition were denoted D1 and D2. These identifiers indicate only the acquisition condition and order; the Training, Calibration, and Test roles varied according to the evaluation direction. Unlike the fixed Training source used for P1, the P2/P3 Training source rotated among the three wearing conditions: both datasets (D1 and D2) from one source condition were combined for Training, while datasets from a different target condition provided Calibration and Test. The two datasets within the same wearing condition were used to evaluate reproducibility between recordings without sensor removal. A classifier was constructed using the two datasets from one wearing condition, and datasets from the other wearing conditions were then used as inputs to evaluate performance degradation associated with sensor re-donning across conditions. In addition, within each target wearing condition, the Calibration and Test roles of the two datasets were exchanged, enabling bidirectional evaluation while keeping the Test data independent from both recalibration and classifier training. The P2/P3 configuration complemented the P1 analysis by evaluating Training-data reuse and performance recovery using two initial recordings from each source wearing condition. The two configurations served complementary purposes rather than a controlled comparison of Training-data volume.

Each dataset was acquired using the same predefined motion protocol. A recording began with a 10-s Rest period. For each finger, a 3-s preparation period and a 3-s countdown were followed by five repetitions of a 3-s finger-flexion period and a 3-s return-to-rest period. The four fingers were measured sequentially in the fixed order of index, middle, ring, and little finger. The same controlled movement protocol was used across wearing conditions to limit variation in task timing and class composition, thereby facilitating interpretation of signal changes associated with sensor re-donning. The sEMG acquisition stream did not itself contain an independent finger-identity label; therefore, the predetermined command order was required to associate each detected motion interval consistently with the intended finger class for quantitative analysis. The fixed order also kept the temporal structure of the Training, Calibration, and Test recordings matched across participants, datasets, and wearing conditions. After completion of the little-finger trials, a final 5-s Rest period was recorded. Therefore, the sequence of each dataset was

Rest→Index→Middle→Ring→Little→Rest.

The nominal duration of one dataset was

$$10+4\left[3+3+5(3+3)\right]+5=159s,$$

corresponding to 20 finger-flexion events per dataset. The recorded durations were approximately 158.6 s, consistent with the nominal protocol duration. Although each dataset included Rest intervals as part of the predefined motion protocol, Rest windows were not used as outputs in the present four-class finger-motion classification or evaluation. Rest was nevertheless retained as a fifth labeled class when estimating the recalibration transformations. Transforming the complete labeled Training signal required valid class-specific statistics for Rest as well as for the four finger motions. In particular, because the causal motion windows could contain pre-onset Rest samples, applying the Rest transformation before window generation allowed those samples to be adapted consistently with the remainder of the Training signal.

### 2.2. Motion-Interval Detection, Labeling, and Window Segmentation

**Figure 3:**
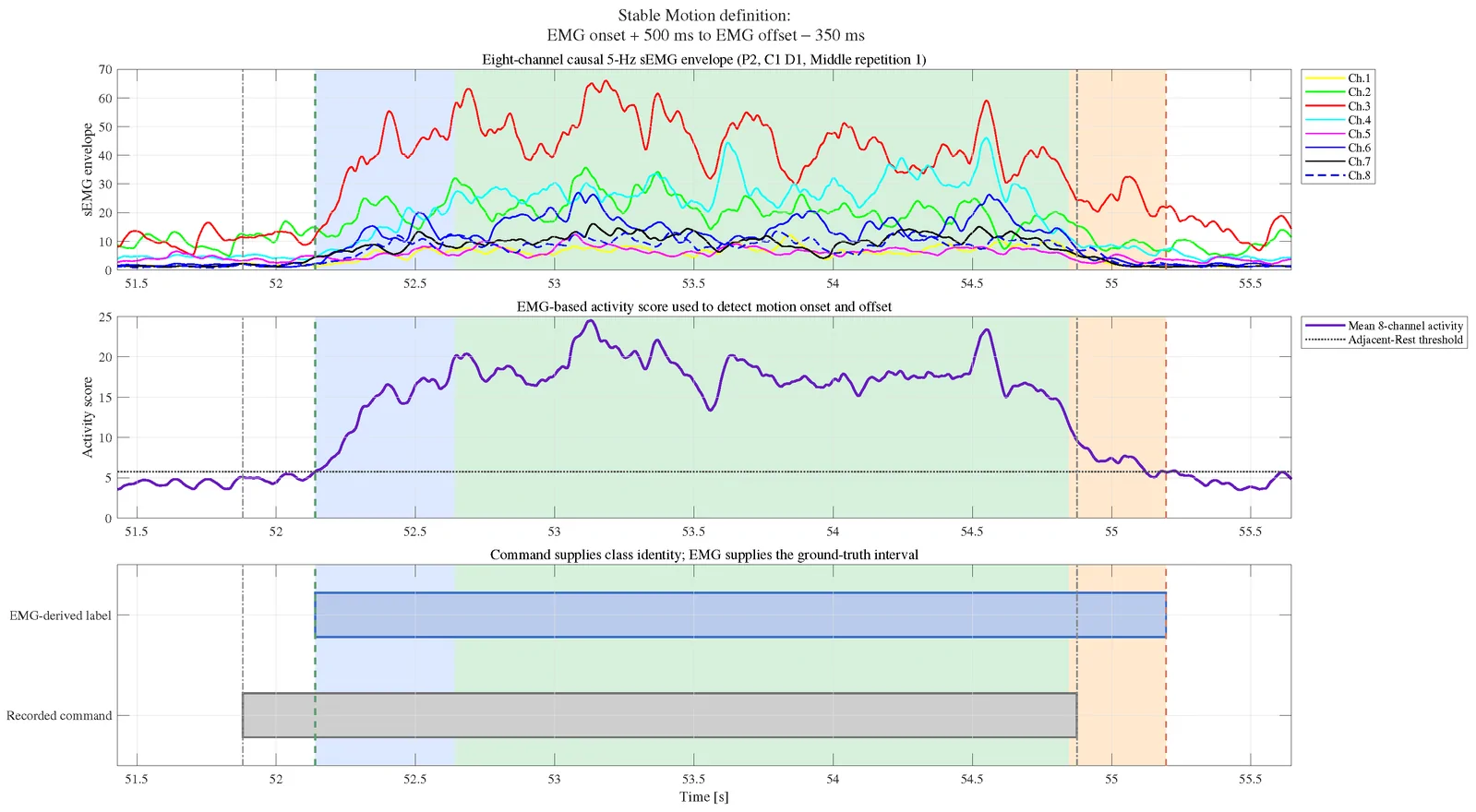
Representative detection of a motion interval and the corresponding Stable Motion region. The upper panel shows the eight-channel sEMG envelopes obtained using a causal 5-Hz low-pass filter. The middle panel shows the mean eight-channel activity score and the threshold derived from adjacent-Rest data. The lower panel compares the recorded command interval with the EMG-derived motion interval. The blue, green, and orange shaded regions denote the onset-exclusion interval, Stable Motion interval, and offset-exclusion interval, respectively.

Motion intervals were detected from the recorded motion command and the mean eight-channel sEMG activity, as illustrated in Figure 3. The command identified the instructed finger and constrained the interval in which onset and offset were searched; it did not directly define those boundaries.

For each command interval, the eight sEMG channels were full-wave rectified and processed using a second-order causal Butterworth low-pass filter with a cutoff frequency of 5 Hz. The filtered channels were then averaged to form a mean eight-channel activity signal. The filter was applied once to each complete recording in the forward direction, with zero initial states at the beginning of the recording and without resetting the filter state for individual command intervals; no bidirectional filtering was used. The 5-Hz cutoff was selected to obtain a smooth rectified-sEMG envelope for threshold-based motion-boundary detection. This value lies within the commonly used 3–10-Hz range for EMG-envelope extraction [15] and, relative to the 3-s contractions and 150-ms persistence criterion used in the present protocol, suppresses rapid EMG fluctuations while retaining task-level changes in muscle activation. The cutoff was fixed across all datasets and evaluation conditions.

For each command interval, the adjacent-Rest reference comprised Rest-labeled samples from 2.0 to 0.2 s before command onset and from 0.2 to 2.0 s after command offset. Samples from the two ranges were combined; if neither range contained Rest samples, all Rest-labeled samples in the recording were used. For an adjacent-Rest activity vector *a*, the robust standard deviation was defined as 1.4826median(|a−median(a)|); the ordinary standard deviation was used as a fallback if this value was non-finite or non-positive. The detection threshold was the larger of the 95th percentile of the adjacent-Rest activity and the adjacent-Rest median plus three robust standard deviations.

The search ranged from command onset to 1 s after command offset. At 200 Hz, the 150-ms persistence interval contained 30 samples. A block qualified as active when at least 60% of its samples exceeded the threshold. Onset was defined as the first threshold-exceeding sample within the first qualifying block, and offset was defined as the last threshold-exceeding sample within the final qualifying block, with one sample interval added to the reported offset time. Events without a valid persistent EMG response were excluded. The 150-ms/60% persistence rule was specified as a fixed engineering criterion to suppress brief threshold excursions and was applied consistently across all analyzed datasets.

Each detected motion interval was assigned the commanded finger class (Index, Middle, Ring, or Little).

Feature extraction and classifier training used the raw signed eight-channel sEMG within these labeled motion intervals, without rectification or envelope filtering. The 5-Hz envelopes were used only for motion-boundary detection and visualization and were not inputs to the four-class ECOC-SVM. No temporal smoothing or prediction correction was applied to the classifier outputs.

Offline processing and analysis were implemented in MATLAB R2024b Update 6 (version 24.2.0.2923080) on Windows 11 Home (verification environment: Build 26200), using Statistics and Machine Learning Toolbox 24.2, Signal Processing Toolbox 24.2, and Optimization Toolbox 24.2.

The sEMG signals were segmented using a causal sliding window of 300 ms, corresponding to 60 samples at the 200-Hz sampling frequency. Consecutive windows were updated every 60 ms, corresponding to 12 samples and an overlap of 80%. Each window was assigned the ground-truth class at its final sample. Therefore, each window contained only the current and preceding samples available at the corresponding window endpoint. The 300-ms/60-ms operating point was selected from a prior Base-session window/update sweep (200/250/300 ms × 50/60/70/80 ms) as a joint setting that maximized or nearly maximized Stable Motion finger-classification and end-to-end class-balanced metrics and was subsequently fixed for all re-donning evaluations.

As illustrated in Figure 3, two temporal criteria were applied to the EMG-derived motion interval. For classifier training, windows whose endpoints occurred from 100 ms after onset to 350 ms before offset were used so that early muscle-activation changes were included in model construction. For evaluation, a stricter Stable Motion interval was used, from 500 ms after onset to 350 ms before offset, in which finger-specific sEMG information was considered sufficiently established. Performance degradation after sensor re-donning and its recovery by recalibration were therefore assessed in this information-rich stable region rather than during the onset and offset transitions. Only Index, Middle, Ring, and Little windows satisfying the corresponding temporal criterion were retained.

### 2.3. Baseline Participant-Specific Classification System

A participant-specific four-class ECOC-SVM finger-motion classifier was constructed using the Training data. The same feature-extraction and classification procedure was used for P1, P2, and P3. The raw signed eight-channel sEMG signals were used to characterize the inter-channel covariance structure. For each 300-ms window represented by $W\in R^{60\times 8}$, the sample covariance matrix was calculated using the N−1 normalization:

(1) C=cov(W).

The covariance matrix was regularized as

(2) $$C_{\mathrm{reg}}=\left(1-\lambda \right)C+\lambda \frac{\mathrm{tr}(C)}{8}I_{8},\;\lambda =0.05,$$

The shrinkage coefficient λ=0.05 was fixed a priori to improve numerical conditioning of the sample covariance before the matrix logarithm and was used consistently across all evaluations. The regularized covariance matrix was subsequently normalized by its trace:

(3) $$\tilde{C}=\frac{C_{\mathrm{reg}}}{\mathrm{tr}(C_{\mathrm{reg}})}.$$

A matrix logarithm was then applied:

(4) $$L=\log(\tilde{C}).$$

In the implementation, the matrix logarithm was calculated through eigenvalue decomposition, with eigenvalues smaller than $10^{-10}$ replaced by $10^{-10}$ before taking the logarithm. This eigenvalue floor was used as a numerical safeguard for the matrix logarithm rather than as a performance-tuned threshold. The upper-triangular elements of L were vectorized, with diagonal elements retained unchanged and off-diagonal elements multiplied by $\sqrt{2}$. Because L is an 8×8 symmetric matrix, this procedure resulted in a 36-dimensional trace-normalized log-covariance feature vector.

For classifier training, each feature vector was standardized using only its corresponding training data. For each feature dimension, the training-set mean and standard deviation were calculated and subsequently applied to both the training and evaluation data. Thus, no held-out Test data were used to estimate the normalization parameters.

The classifier was a four-class linear error-correcting output codes (ECOC) SVM with one-versus-one coding for classification of the Index, Middle, Ring, and Little fingers. It used a box constraint of 1 and uniform class priors, without additional sample weighting or class-dependent cost adjustment.

All classifier and preprocessing parameters described above were fixed across participants and evaluation conditions before held-out Test evaluation.

Training windows were selected using the EMG-derived motion intervals and the 100 ms after onset to 350 ms before offset criterion defined in Section 2.2.

### 2.4. Proposed Personalized Recalibration Framework

Sensor re-donning can alter the measurement characteristics of multi-channel sEMG signals relative to those represented by the Training data. Because these changes are not necessarily represented by uniform scaling across all channels and classes, amplitude-only correction using a single set of channel-wise gains shared across classes may be insufficient to compensate for the distribution mismatch between the Training-data condition and the current sensor-wearing condition. In addition to changes in channel amplitudes, sensor re-donning may also alter the relative channel-activation patterns and inter-channel covariance structures of the measured sEMG signals.

Based on this consideration, the recalibration problem in this study was formulated as adaptation of the Training-data distribution rather than restoration of the current sEMG signals to the previous measurement condition. After sensor re-donning, a small amount of labeled sEMG data acquired under the current sensor-wearing condition was used as Calibration data. These data served as a reference for transforming the participant-specific Training data toward the current measurement condition. Thus, the direction of adaptation was from the Training-data condition toward the current condition rather than from the current signals toward the Training-data condition.

The proposed recalibration procedure consists of two successive adaptation steps. First, classwise amplitude correction is applied to the Training signals using class- and channel-specific amplitude characteristics estimated from both the Training data and the current Calibration data. Second, classwise covariance alignment is applied to the amplitude-corrected Training signals to reduce the mismatch between their inter-channel covariance structures and those estimated from the corresponding classes in the current Calibration data. Importantly, the source covariance for this transformation is estimated from the amplitude-corrected Training data rather than from the original Training data.

After these adaptation steps, the 36-dimensional trace-normalized log-covariance features described in Section 2.3 are re-extracted from the Adapted training data, and the four-class ECOC-SVM finger-motion classifier is retrained. The same recalibration and retraining procedure is applied to P1, P2, and P3. As an order-of-magnitude indication rather than a deployment benchmark, the combined offline pipeline was timed 20 times for Participant 1 on a representative Calibration–Test fold (test3, fold 1; five Training datasets) on an 11th Gen Intel Core i5-1135G7 CPU (4 cores/8 logical processors; MATLAB R2024b Update 6). Each timed repetition included Calibration amplitude and covariance statistics, classwise amplitude and covariance adaptation of the five Training datasets, 36-dimensional feature re-extraction, and ECOC-SVM retraining on 4370 training windows. The first timed repetition required 3.50 s (adaptation 0.16 s, feature re-extraction 0.75 s, and retraining 2.59 s). Over the subsequent 19 repetitions, the wall-clock time was 0.81±0.31 s (adaptation 0.10±0.04 s, feature re-extraction 0.49±0.18 s, and retraining 0.22±0.10 s; mean ± std). File input/output, label generation, construction of the fixed Training amplitude reference, MATLAB startup, Calibration acquisition, and Test prediction were excluded; the runs were not optimized for latency.

The Test data remain unchanged throughout the recalibration procedure. They are not used to estimate the amplitude-correction parameters, covariance-alignment parameters, feature-standardization parameters, or classifier parameters, and are used only to evaluate the recalibrated classifier. The overall procedure can therefore be summarized as

TrainingData→CurrentCalibrationAmplitudeCorrectionAmplitude-CorrectedTrainingData→CurrentCalibrationClasswiseCovarianceAlignmentAdaptedTrainingData→FeatureRe-extraction→SVMRetraining.

The detailed procedures for classwise amplitude correction and classwise covariance alignment are described in the following subsections.

### 2.5. Classwise Amplitude Correction

The first recalibration step adjusts the class- and channel-dependent amplitude characteristics of the Training data toward those observed in the current Calibration data. Because the amplitude changes after sensor re-donning may differ among both classes and channels, the proposed method estimates an independent gain for each class–channel pair rather than applying a single channel-wise gain pattern to all classes.

The reference amplitude was estimated from the raw eight-channel sEMG signals without envelope filtering. The signals were segmented using the same 300-ms windows and 60-ms update interval described in Section 2.2. Only amplitude-reference windows were used for reference estimation. An amplitude-reference window was defined as a window whose endpoint was located at least 500 ms after the onset and at least 500 ms before the offset of the corresponding class interval, and whose entire 300-ms interval belonged to the same class. For Rest, each contiguous Rest-labeled segment was treated as a class interval, so a Rest amplitude-reference window had to lie entirely within that segment, with its endpoint at least 500 ms after the Rest-segment onset and at least 500 ms before its offset. This restriction excluded windows near motion offset, in which declining muscle activation could otherwise reduce the estimated representative amplitude.

For each Training dataset *s*, class *c*, and channel *i*, the root mean square (RMS) value of each amplitude-reference window *w* was calculated as

(5) $$R_{w,c,i}^{\left(s\right)}=\sqrt{\frac{1}{N}\sum_{n=1}^{N}{\left(x_{w,c,i}^{\left(s\right)}\left[n\right]\right)}^{2}},$$

where N=60 is the number of samples in a 300-ms window. Let $W_{s,c}$ denote the set of amplitude-reference windows belonging to class *c* in the training dataset *s*. The dataset-specific representative amplitude for each class–channel pair was defined as the median of the window-wise RMS values:

(6) $$A_{c,i}^{\left(s\right)}={\mathrm{median}}_{w\in W_{s,c}}R_{w,c,i}^{\left(s\right)}.$$

For the Training-data condition, the reference amplitude was obtained by averaging the corresponding representative amplitudes across the Training datasets available for participant *p*:

(7) $$A_{c,i}^{\mathrm{Train}}=\frac{1}{B_{p}}\sum_{s=1}^{B_{p}}A_{c,i}^{\left(s\right)}.$$

where $B_{p}$ denotes the number of Training datasets available for participant *p*. The Calibration reference amplitude, $A_{c,i}^{\mathrm{Cal}}$, was calculated directly as the median window-wise RMS value from the current Calibration dataset using the same procedure.

The class- and channel-specific amplitude gain was defined as

(8) $$g_{c,i}=\frac{A_{c,i}^{\mathrm{Cal}}}{A_{c,i}^{\mathrm{Train}}},\;c=0,\ldots ,4,\;i=1,\ldots ,8.$$

Thus, a 5×8 gain matrix was obtained for the five classes (Rest, Index, Middle, Ring, and Little) and eight sEMG channels. No upper or lower clipping was applied to the estimated gains, and all reference amplitudes and gains were required to be positive and finite.

Rest was included because the transformation was applied sample-wise to the complete labeled Training signal before causal windows were generated. Consequently, Rest statistics were required to adapt both the explicit Rest intervals and the pre-onset Rest samples contained in early motion windows, even though the final classifier produced only the four finger-motion classes.

The gain matrix was applied sample-wise to the Training signals. For a Training sample $x_{t}$ belonging to class $y_{t}$, the amplitude-corrected signal was calculated as

(9) $${\tilde{x}}_{t}=g_{y_{t}}⊙x_{t},$$

where $g_{y_{t}}\in R^{8}$ denotes the channel-wise gain vector corresponding to class $y_{t}$, and ⊙ denotes element-wise multiplication. Therefore, each class was transformed using a different eight-channel gain pattern. Because this transformation was applied exclusively to the labeled Training data, the class label required to select the corresponding gain vector was already available. No class information from the Test data was required.

The Calibration data were used only to estimate the Calibration reference amplitudes, whereas the Training signals were the data directly transformed by the proposed correction. The Test data were not used to estimate the reference amplitudes or gains and were not modified by the amplitude-correction procedure. After amplitude correction, the trace-normalized log-covariance features were re-extracted, and the four-class ECOC-SVM was retrained. The amplitude-corrected Training signals were subsequently used as the source data for the classwise covariance-alignment procedure described in the following subsection.

### 2.6. Classwise Covariance Alignment

Following classwise amplitude correction, classwise covariance alignment was applied to further adapt the inter-channel structure of the Training signals toward that observed in the current Calibration data. Whereas the amplitude correction described in the previous subsection adjusts class- and channel-dependent signal magnitudes, the covariance-alignment step accounts for differences in the joint variation among the eight sEMG channels.

Unlike the reference-amplitude estimation described in Section 2.5, no amplitude-reference window restriction was applied when estimating the class means and covariance matrices. For each Training dataset *s*, all raw sEMG samples assigned to each class were used to estimate the corresponding source statistics. For Rest, this included all Rest-labeled raw samples from the initial, inter-motion, and final Rest periods, including transition samples that remained labeled as Rest. Consequently, samples near motion transitions could also contribute to these estimates. The target statistics were estimated in the same manner using all raw samples assigned to each class in the labeled current Calibration dataset.

For a given dataset and class *c*, let $X_{c}\in R^{N_{c}\times 8}$ denote the raw eight-channel sEMG samples assigned to that class. The class mean was calculated as

(10) $$\mu_{c}=\frac{1}{N_{c}}\sum_{n=1}^{N_{c}}x_{c,n},$$

and the empirical covariance matrix was calculated using normalization by $N_{c}$:

(11) $$S_{c}=\frac{1}{N_{c}}\sum_{n=1}^{N_{c}}\left(x_{c,n}-\mu_{c}\right){\left(x_{c,n}-\mu_{c}\right)}^{T}.$$

To improve numerical stability and obtain a well-conditioned positive-definite covariance matrix, shrinkage regularization was applied as

(12) $$C_{c}=\left(1-\alpha \right)S_{c}+\alpha \frac{\mathrm{tr}(S_{c})}{8}I,$$

where α=0.05 and I denotes the 8×8 identity matrix. The covariance matrix was symmetrized before regularization. After regularization, it was reconstructed from its eigendecomposition with eigenvalues smaller than $10^{-10}$ replaced by $10^{-10}$ and was symmetrized again. As in the baseline feature extraction, α=0.05 and the $10^{-10}$ eigenvalue floor were fixed a priori as numerical-stability choices and applied consistently across all evaluations.

In the combined amplitude–covariance procedure, the source statistics were estimated separately for each amplitude-corrected Training dataset. Let $\mu_{s,c}^{\mathrm{src}}$ and $C_{s,c}^{\mathrm{src}}$ denote the mean and regularized covariance matrix of class *c* in amplitude-corrected Training dataset *s*, respectively. Let $\mu_{c}^{\mathrm{Cal}}$ and $C_{c}^{\mathrm{Cal}}$ denote the corresponding target statistics estimated from the labeled current Calibration dataset. The same Calibration statistics served as the target for all Training datasets.

A whitening–coloring transformation was constructed separately for each Training dataset and class pair:

(13) $$A_{s,c}={\left(C_{c}^{\mathrm{Cal}}\right)}^{1/2}{\left(C_{s,c}^{\mathrm{src}}\right)}^{-1/2}.$$

For a source sample x belonging to class *c* in Training dataset *s*, the adapted signal was obtained as

(14) $$x^{\prime }=A_{s,c}\left(x-\mu_{s,c}^{\mathrm{src}}\right)+\mu_{c}^{\mathrm{Cal}}.$$

The matrix square root and inverse square root were calculated by eigendecomposition of the corresponding regularized covariance matrices, with the same eigenvalue floor of $10^{-10}$. This transformation centers and whitens each source class distribution using the statistics of its own Training dataset and subsequently colors and shifts it according to the covariance and mean of the corresponding Calibration class. Thus, the Training datasets were transformed separately rather than being pooled before estimation of the source statistics.

In the proposed combined procedure, classwise amplitude correction was first applied to the Training signals. The class means and covariance matrices were then recalculated from each amplitude-corrected Training dataset and used as the source statistics for covariance alignment. Thus, covariance alignment was estimated after, rather than before, amplitude correction. In the covariance-only comparison condition, the source statistics were instead estimated separately from each original Training dataset, and the same covariance-alignment procedure was applied without the preceding classwise amplitude correction.

The Test data were not used to estimate the class means, covariance matrices, or transformation matrices and were not transformed by the covariance-alignment procedure. Because the class-specific transformations were applied only to labeled Training data during recalibration, ground-truth class information was not required during testing. Following covariance alignment, the log-covariance features were re-extracted, and the four-class ECOC-SVM was retrained.

### 2.7. Compared Methods

All comparison methods used the same ground-truth generation procedure, analysis-window configuration, log-covariance feature definition, four-class ECOC-SVM architecture, and feature-standardization procedure described in Section 2.2 and Section 2.3. A 300-ms analysis window with a 60-ms update interval was used in all conditions. The same Calibration–Test split was maintained across all comparison methods.

For the main conditions involving classifier retraining, the log-covariance features were re-extracted from the corresponding training data, and the four-class ECOC-SVM was retrained. Feature-standardization parameters were estimated using only the corresponding training data. Except for the simplified Test-side correction conditions described below, the Test data were not transformed. In all conditions, the Test data were not used to estimate recalibration parameters, feature-standardization parameters, or classifier parameters. No temporal smoothing, majority voting, hysteresis, or prediction correction was applied in any condition.

The main comparison conditions differed in the direction and type of signal adaptation, the use of covariance alignment, and whether the labeled Calibration data were directly included in classifier training. These conditions are summarized in Table 1.

The Fixed classifier condition served as the baseline. The participant-specific classifier trained using the original Training data was applied directly to the Test data without recalibration or classifier retraining.

Two Common8 conditions were included to examine the effect of a class-independent channel-wise amplitude correction. For each channel, the Training and Calibration reference amplitudes defined in Section 2.5 were first averaged across the five classes, including Rest:

(15) $${\bar{A}}_{i}^{\mathrm{Train}}=\frac{1}{5}\sum_{c=0}^{4}A_{c,i}^{\mathrm{Train}},\;{\bar{A}}_{i}^{\mathrm{Cal}}=\frac{1}{5}\sum_{c=0}^{4}A_{c,i}^{\mathrm{Cal}}.$$

The corresponding Training-to-Calibration and Calibration-to-Training gains were defined as

(16) $$g_{i}^{\mathrm{Train}\to \mathrm{Cal}}=\frac{{\bar{A}}_{i}^{\mathrm{Cal}}}{{\bar{A}}_{i}^{\mathrm{Train}}},\;g_{i}^{\mathrm{Cal}\to \mathrm{Train}}=\frac{{\bar{A}}_{i}^{\mathrm{Train}}}{{\bar{A}}_{i}^{\mathrm{Cal}}}.$$

The resulting channel-wise gain vector was class-independent and was shared across all five classes. In the Common8 + Fixed condition, the Test signals were transformed toward the Training-data amplitude scale using $g_{i}^{\mathrm{Cal}\to \mathrm{Train}}$, while the original fixed classifier was retained without retraining. This condition therefore represented a current-to-historical signal-correction strategy. Because the same eight-channel gain vector was applied to every Test sample regardless of class, this transformation did not require ground-truth class labels from the Test data.

In contrast, in the Common8 retrain condition, the Training signals were transformed toward the current Calibration amplitude scale using $g_{i}^{\mathrm{Train}\to \mathrm{Cal}}$. The log-covariance features were then re-extracted from the amplitude-corrected Training signals, and the four-class ECOC-SVM was retrained.

The Classwise amplitude condition applied only the class- and channel-specific amplitude correction described in Section 2.5, without covariance alignment. The Covariance condition applied only the classwise covariance-alignment procedure described in Section 2.6 to the original Training signals without the preceding classwise amplitude correction. The Classwise amplitude + Covariance condition corresponded to the complete proposed recalibration procedure, in which classwise amplitude correction was first applied to the Training signals and classwise covariance alignment was subsequently applied using the amplitude-corrected Training signals as the source.

For each of these recalibration strategies, an additional +Cal condition was evaluated in which the labeled Calibration data were directly included in the training set together with the Adapted training data. The Calibration signals included in the +Cal conditions were used in their original form and were not subjected to amplitude correction or covariance alignment. These conditions were included to examine whether Calibration data needed to be directly incorporated into classifier training or whether they could instead be used only as a reference for producing the Adapted training data.

Finally, in the Calibration only condition, the Training data were excluded, and the four-class ECOC-SVM was trained using only the labeled Calibration data. This condition was included to compare the reuse of adapted training data with the construction of a new classifier using only the available Calibration data. Within each target wearing condition, all Calibration-using comparison methods were assigned one current-condition recording as Calibration while the paired recording was held out as an independent Test dataset; the Calibration and Test roles were then exchanged. Calibration-only retraining constructed a classifier directly from that single current-condition recording. In the principal Training-data-reuse conditions, the same Calibration recording instead supplied the amplitude and covariance targets used to adapt the historical Training recordings (five for P1 and two for P2/P3), and the classifier was retrained on the resulting Adapted training data. Consequently, the number of classifier-training recordings and windows was intentionally not matched: the comparison evaluated complete recalibration strategies under the same one-recording current Calibration burden, rather than isolated algorithmic effects under matched historical training volume. Calibration-only therefore served as a descriptive within-wearing reference, and the question was whether historical data acquired under another wearing condition could be recalibrated to approach that reference, not whether adaptation was intrinsically superior under equal training-set size. Although the same Calibration recording and selected repetitions were supplied to all methods, their algorithmic use also differed: amplitude correction used edge-restricted RMS windows, covariance alignment used all class-assigned raw samples to estimate target statistics, and Calibration-only retraining used only eligible four-class feature windows as direct classifier-training observations.

Table 2 provides a descriptive accounting of these data uses for the full five-repetition Calibration condition.

To place the proposed signal-space recalibration in the context of a publicly established classical domain-adaptation baseline, correlation alignment (CORAL) [16] was additionally evaluated on the same Calibration–Test splits. Unlike the proposed amplitude and covariance procedures, which transform raw eight-channel Training signals before feature extraction, CORAL was applied in the already extracted 36-dimensional log-covariance feature space. Let $X^{\mathrm{src}}$ and $X^{\mathrm{Cal}}$ denote the Training and Calibration feature matrices (rows as windows). With source and Calibration means $\mu_{s}$ and $\mu_{t}$ and regularized covariances $C_{s}$ and $C_{t}$, the CORAL map was

(17) $$X^{\prime }=\left(X^{\mathrm{src}}-\mu_{s}\right)C_{s}^{-1/2}C_{t}^{1/2}+\mu_{t}.$$

In the Global CORAL condition, $C_{s}$ and $C_{t}$ were estimated from all eligible four-class classifier-training feature windows pooled across the finger classes, providing a standard unsupervised CORAL reference. In the Classwise CORAL condition, the same transformation was estimated separately for each finger class using the eligible labeled classifier-training feature windows, which is the more directly comparable supervised reference under the present multi-class protocol. Adapted Training features were then used to retrain the four-class ECOC-SVM; Test features were not transformed. Global CORAL is reported as a classical unsupervised reference rather than as the primary competitor to the proposed method, because a single pooled covariance combines within-class and between-class structure across the four finger classes.

Separately from the main comparison conditions summarized in Table 1, a simplified fixed-model analysis was conducted to determine whether recalibration could be achieved by correcting only the incoming Test signals toward the Training-data condition. This analysis retained the original fixed four-class ECOC-SVM and its feature-standardization parameters without retraining. In addition to the Fixed classifier and Common8 + Fixed conditions defined above, two class-independent Test-side distribution-correction conditions were evaluated.

For this analysis, all raw samples assigned to the Index, Middle, Ring, and Little classes were pooled across the five Training datasets, while Rest samples were excluded. Let $\mu_{G}^{\mathrm{Train}}$ and $C_{G}^{\mathrm{Train}}$ denote the mean and regularized covariance matrix of these pooled Training motion samples. The corresponding statistics estimated from the labeled current Calibration dataset were denoted by $\mu_{G}^{\mathrm{Cal}}$ and $C_{G}^{\mathrm{Cal}}$. The covariance matrices were regularized using the procedure described in Section 2.6.

In the GlobalCov + Fixed condition, a single Calibration-to-Training whitening–coloring transformation was calculated as

(18) $$A_{\mathrm{Cal}\to \mathrm{Train},G}={\left(C_{G}^{\mathrm{Train}}\right)}^{1/2}{\left(C_{G}^{\mathrm{Cal}}\right)}^{-1/2}.$$

The corrected Test signal was then obtained as

(19) $$x^{\prime }=A_{\mathrm{Cal}\to \mathrm{Train},G}\left(x-\mu_{G}^{\mathrm{Cal}}\right)+\mu_{G}^{\mathrm{Train}}.$$

The same transformation was applied to every Test sample and therefore did not require the true finger class of the Test sample.

The Common8 + GlobalCorr + Fixed condition first applied the Calibration-to-Training Common8 gains defined above to both the Calibration and Test signals. To avoid overwriting the channel-amplitude scaling introduced by Common8, the subsequent alignment was performed using correlation rather than covariance matrices. Let $R_{G}^{\mathrm{Train}}$ denote the correlation matrix of the pooled Training motion samples, and let $R_{G}^{\mathrm{Cal},C8}$, $\mu_{G}^{\mathrm{Cal},C8}$, and $D_{G}^{\mathrm{Cal},C8}$ denote the correlation matrix, mean, and diagonal standard-deviation matrix of the Common8-corrected Calibration motion samples. The correlation alignment was calculated as

(20) $$A_{\mathrm{Corr},G}={\left(R_{G}^{\mathrm{Train}}\right)}^{1/2}{\left(R_{G}^{\mathrm{Cal},C8}\right)}^{-1/2},$$

and applied to each Common8-corrected Test sample $x_{C8}$ as

(21) $$x^{\prime }=D_{G}^{\mathrm{Cal},C8}A_{\mathrm{Corr},G}{\left(D_{G}^{\mathrm{Cal},C8}\right)}^{-1}\left(x_{C8}-\mu_{G}^{\mathrm{Cal},C8}\right)+\mu_{G}^{\mathrm{Cal},C8}.$$

Thus, the Common8-corrected channel means and variances were retained, while the pooled inter-channel correlation structure was mapped toward that of the Training data. In both global alignment conditions, the correction parameters were estimated exclusively from the Training and Calibration data. The Test data were not used to estimate means, covariance or correlation matrices, gains, feature-standardization parameters, or classifier parameters.

### 2.8. Calibration Repetition-Count Analysis Protocol

An exploratory analysis was performed to examine whether reusing previously acquired Training data could reduce the amount of newly acquired Calibration data required after sensor re-donning. All original recordings followed the full protocol of five flexion repetitions for each of the Index, Middle, Ring, and Little fingers. Shorter Calibration conditions were therefore constructed retrospectively from these recordings rather than acquired as separate shorter datasets.

For each Calibration dataset, the first r∈{1,2,3,5} valid repetitions of each finger were retained in temporal order. The five-repetition condition used all available repetitions and served as Full Calibration. At each value of *r*, exactly the same selected Calibration subset was supplied to all compared methods, while the paired Test dataset remained unchanged and was excluded from parameter estimation and classifier training. Three conditions were compared: a new four-class ECOC-SVM trained using only the selected Calibration repetitions (Calibration only), covariance-based adaptation and reuse of the Training data, and combined amplitude–covariance adaptation and reuse of the Training data. In the two reuse conditions, the selected repetitions were used to estimate the current-wearing adaptation parameters; the classifier itself was retrained using the resulting Adapted training data.

Under the nominal acquisition schedule defined in Section 2.1, retaining the initial and final Rest periods, the preparation and countdown intervals, and a 3-s flexion followed by a 3-s return period for each repetition, the duration of a shortened Calibration recording would be

$$T\left(r\right)=10+4\left[3+3+r(3+3)\right]+5=39+24rs.$$

The nominal durations for one, two, three, and five repetitions per finger are therefore 63, 87, 111, and 159 s, respectively. In particular, reducing the Calibration protocol from five to two repetitions per finger would correspond to a theoretical reduction of 72 s, or approximately 45%, if the same timing structure were retained. These durations were calculated from the protocol timing; separate prospectively shortened Calibration recordings were not acquired.

To assess sensitivity to repetition selection, an exhaustive supporting analysis evaluated all shared repetition-index subsets of size r∈{1,2,3,5} for P1–P3 using the same Combined amplitude–covariance pipeline. The same repetition indices were applied to every finger class (51+52+53+55=26 subsets per Calibration–Test fold). P1 contributed ten folds, whereas P2 and P3 contributed 12 folds each (six directed wearing-condition pairs × two Calibration–Test exchanges), including all six directed pairs rather than only the post hoc failure-case subsets. For each subset, Calibration amplitude and covariance statistics were re-estimated, the Training data were adapted, and Stable Motion accuracy was calculated on the unchanged Test dataset. Subset outcomes were first averaged within each fold and then summarized across folds because subsets sharing repetitions were not independent. One P1 single-repetition subset (test4, fold 1, repetition 5) was unavailable because Little-finger Calibration samples were missing; no P2/P3 subsets were excluded. Signal-level quantities additionally included class–channel amplitude gains and classwise log-Euclidean covariance distances. For P1 under full five-repetition Calibration, the covariance-distance analysis included within-wearing references (pairwise distances among the five Training datasets and Calibration–Test distances within each re-donning condition), the Training–Test distance before adaptation, and three distances after Combined adaptation (among Adapted training datasets, Adapted training–Calibration, and Adapted training–Test). Distances were first averaged over Rest and the four finger classes. Euclidean distances between classwise 36-dimensional log-covariance feature centroids were also calculated for Training versus Calibration and Adapted training versus Calibration, together with subset-versus-full-five log-gain and Calibration covariance deviations. All three participants followed the same measurement protocol, including the same armband acquisition configuration, predefined finger-command order, and five-repetition structure.

### 2.9. Evaluation Protocol and Metrics

All evaluation protocols used the comparison conditions defined in Section 2.7, unless otherwise stated. The Test dataset was kept completely separate from recalibration-parameter estimation and classifier training. It was not used to estimate amplitude-correction gains, class means, covariance or correlation matrices, feature-standardization parameters, or classifier parameters. For the main recalibration comparisons involving Training-data adaptation and classifier retraining, the Test signals remained unchanged. Test-signal transformation was performed only in the separate simplified fixed-model analysis described in Section 2.7. Feature standardization was performed using parameters estimated exclusively from the corresponding training data.

The held-out signals were segmented using the same 300-ms causal windows described in Section 2.2. The primary outcome was Stable Motion window-level four-finger classification performance, using the motion-interval detection, labeling, and Stable Motion definition given in that section.

The participant-specific Training and re-donning configurations described in Section 2.1 were retained. The calibration-amount comparisons followed Section 2.8. P1, P2, and P3 shared the same Stable Motion four-finger endpoint.

Log-covariance features and amplitude-reference and covariance statistics were calculated from raw signed sEMG as described in Section 2.2, Section 2.3, Section 2.5 and Section 2.6.

For a *K*-class confusion matrix $M\in R^{K\times K}$, let $M_{i,j}$ denote the number of windows with ground-truth class *i* predicted as class *j*. Classification accuracy was calculated as

$$\mathrm{Accuracy}=\frac{\sum_{k=1}^{K}M_{k,k}}{\sum_{i=1}^{K}\sum_{j=1}^{K}M_{i,j}}.$$

For class *k*, recall was calculated as

$${\mathrm{Recall}}_{k}=\frac{M_{k,k}}{\sum_{j=1}^{K}M_{k,j}}.$$

Here, K=4 for the Stable Motion four-finger evaluation. Unless otherwise stated, every subsequently reported accuracy value is this window-level overall accuracy, i.e., the proportion of correctly classified Stable Motion windows across the four finger classes. Classwise performance is reported as recall. Because the number of eligible Stable Motion windows can differ slightly among finger classes and evaluation units, balanced accuracy (the unweighted mean of the four classwise recalls) and macro-averaged F1-score were additionally reported for the main pooled comparisons. The total number of misclassified windows was also recorded. Performance metrics were first calculated separately for each held-out evaluation direction. For aggregate evaluation, window-level predictions from the corresponding Test datasets were pooled before calculating the confusion matrix and performance metrics. Thus, a pooled accuracy value uses the same window-level overall accuracy definition, but is computed after combining the eligible windows from the indicated evaluation directions. Pooled accuracy was retained as the primary descriptive endpoint for continuity with the main Results tables, but it was complemented by evaluation-unit summaries of cross-dataset or cross-direction variability and by balanced accuracy and macro-F1. Because consecutive windows overlapped by 80%, the individual Stable Motion windows were treated as descriptive observations rather than as independent statistical units. Method comparisons and uncertainty summaries were therefore additionally reported at the evaluation-unit level used in the cross-participant paired method-difference analysis: the ten held-out Test datasets for P1 and the six directed wearing-condition pairs for P2 and P3. For each method, the unweighted mean, sample standard deviation, median, interquartile range, and range of the unit-level accuracies were calculated. An evaluation-unit bootstrap interval for the mean unit-level accuracy was obtained by resampling evaluation units with replacement (B=10,000; seed 20260930; percentile 95% interval). Overlapping windows were not resampled independently, and no window-level *p*-values were computed. Because the directed pairs reuse recordings across source and target assignments and the number of evaluation units was small, these bootstrap intervals were interpreted as descriptive within-participant uncertainty summaries rather than population-level inferential intervals.

#### 2.9.1. P1: Five-Dataset Training and Multi-Condition Re-Donning Evaluation

P1 contributed 15 datasets in total. Five datasets were first recorded under one initial wearing condition without removing or repositioning the sensor and were used as the Training data for the participant-specific classifier. Within-wearing discriminability was assessed by leave-one-dataset-out validation: in each fold, four datasets were used for classifier construction, and the remaining dataset was held out for evaluation. The fixed classifier used in the subsequent re-donning analysis was then constructed using all five Training datasets.

The remaining ten datasets were acquired under five separate re-donning conditions, with two consecutive datasets recorded under each condition. For each re-donning condition, one dataset supplied the labeled Calibration data and the other was held out as Test data; their roles were then reversed. The original five Training datasets remained the source data in both directions. The fixed-classifier condition evaluated each Test dataset without adaptation, whereas the recalibration conditions used only its paired Calibration dataset to adapt the Training data before retraining and evaluation.

This configuration established an initial model supported by multiple same-wearing Training datasets and evaluated it across a relatively broad set of independently re-donned wearing conditions. It was intended to determine how much a classifier that performed reliably under its original wearing condition could deteriorate across subsequent wearing states, how variable that deterioration was, and whether the proposed Training-data reuse restored Stable Motion four-finger accuracy. P1 also supported the exploratory Calibration-repetition analysis in Section 2.8 and the simplified fixed-model comparison in Section 2.7.

#### 2.9.2. P2 and P3: Three-Condition Rotating Evaluation with Six Datasets

P2 and P3 each contributed six datasets, comprising D1 and D2 acquired consecutively under each of the three wearing conditions C1–C3. The same six datasets were assigned different roles according to the evaluation direction. First, same-wearing reproducibility was assessed separately within each condition by training on one dataset and testing on its paired dataset, followed by reversal of these roles. This analysis examined whether a pattern learned from one recording remained reproducible in another recording obtained without sensor removal or repositioning.

For the cross-wearing analysis, the two datasets from one wearing condition were combined and used as the Training data. The resulting fixed classifier was evaluated on all four datasets from the other two wearing conditions. Each of the three conditions served in turn as the Training condition, allowing all directed source-to-target wearing comparisons to be examined. For recalibration within a target condition, one of its two datasets was used as Calibration data and the paired dataset was held out as Test data; their roles were then reversed. The Test dataset was excluded from adaptation, standardization, and classifier training in every direction.

This symmetric design examined the same degradation-and-recovery question as P1 with fewer initial recordings. It tested whether a classifier constructed from two same-wearing datasets remained accurate for a compatible wearing distribution, how severely it deteriorated for a shifted distribution, and whether adapting and reusing those two Training datasets restored performance. Because each condition could serve as Training, Calibration, or Test data in different held-out directions, the design also distinguished wearing pairs for which recalibration offered little benefit from those for which it was needed.

## 3. Results

The primary results address four successive questions: how accurately the four finger motions were discriminated within the Training-data wearing condition, how this discrimination changed after sensor re-donning when the classifier was kept fixed, whether the proposed recalibration recovered the lost performance, and how the amount of Calibration data affected the observed performance of complete Training-data-reuse and Calibration-only strategies. These questions are evaluated using Stable Motion window-level four-finger classification. P1 is first evaluated using five same-wearing Training datasets and ten Test datasets obtained under five re-donning conditions, including the corresponding exploratory analysis of Calibration repetition count. P2 and P3 are subsequently evaluated using the three-condition rotating evaluation with six datasets, including their respective exploratory Calibration-repetition analyses. A supporting analysis of simplified Test-side correction while retaining the fixed classifier is reported after these participant-specific results.

### 3.1. Participant 1: Five-Dataset Training and Multi-Condition Re-Donning Evaluation

#### 3.1.1. Within-Training Stable-Motion Classification Performance

Table 3 presents the within-Training leave-one-dataset-out Stable Motion window-level four-finger results for Participant 1. The Stable Motion evaluation region was defined using the common procedure illustrated in Figure 3.

The accuracy was 100.00%, 100.00%, 100.00%, 99.47%, and 99.32% for Training datasets 1–5, respectively. Across the five held-out Training datasets, only 9 of 3707 Stable Motion windows were misclassified, corresponding to a pooled accuracy of 99.76%. Thus, the four finger motions remained highly discriminable across all five held-out Training datasets acquired without changing the sensor-wearing condition. Of the four errors in Training dataset 4, three were Index-to-Middle confusions, and one was an Index-to-Little confusion. Of the five errors in Training dataset 5, two were Middle-to-Little confusions, and three were Little-to-Index confusions. This near-perfect performance under the unchanged sensor-wearing condition provided a reference for determining how much classification accuracy decreased after sensor re-donning.

#### 3.1.2. Stable-Motion Classification After Sensor Re-Donning

The fixed four-class ECOC-SVM trained using the five Training datasets was applied without recalibration or retraining to the two datasets acquired under each of the five sensor re-donning conditions. The paired datasets are denoted as Dataset 1 and Dataset 2 according to their acquisition order. Because the Fixed classifier condition did not use Calibration data, the two evaluation directions within each re-donning condition corresponded simply to evaluating each paired dataset as the Test dataset. Table 4 presents the resulting Stable Motion window-level four-finger performance.

Stable Motion accuracy remained at 100.00% for both datasets in re-donning condition 1. High accuracy was also retained in conditions 4 and 5, ranging from 93.50% to 100.00%. In contrast, accuracy decreased to 83.99% and 84.63% for the two datasets in condition 2 and to 66.58% and 69.85% for those in condition 3. Thus, sensor re-donning did not invariably degrade stable finger-motion classification; rather, the magnitude of degradation depended strongly on the resulting sensor-wearing condition. The two datasets acquired consecutively under each re-donning condition showed broadly similar performance patterns, with a maximum within-condition difference of 4.26 percentage points. These dataset-wise differences illustrate that a single pooled accuracy can mask substantial cross-condition variation; evaluation-unit summaries are therefore reported in the cross-participant comparison.

The degradation was also class-dependent. Ring-motion recall remained 100.00% in all ten held-out datasets. In condition 2, Middle-motion recall decreased to 44.79% and 45.16%. In condition 3, Middle-motion recall further decreased to 17.01% and 2.58%, while Index-motion recall decreased to 52.02% and 77.16%. Across the ten datasets, the dominant error types were Middle-to-Little and Index-to-Little confusions.

**Figure 4:**
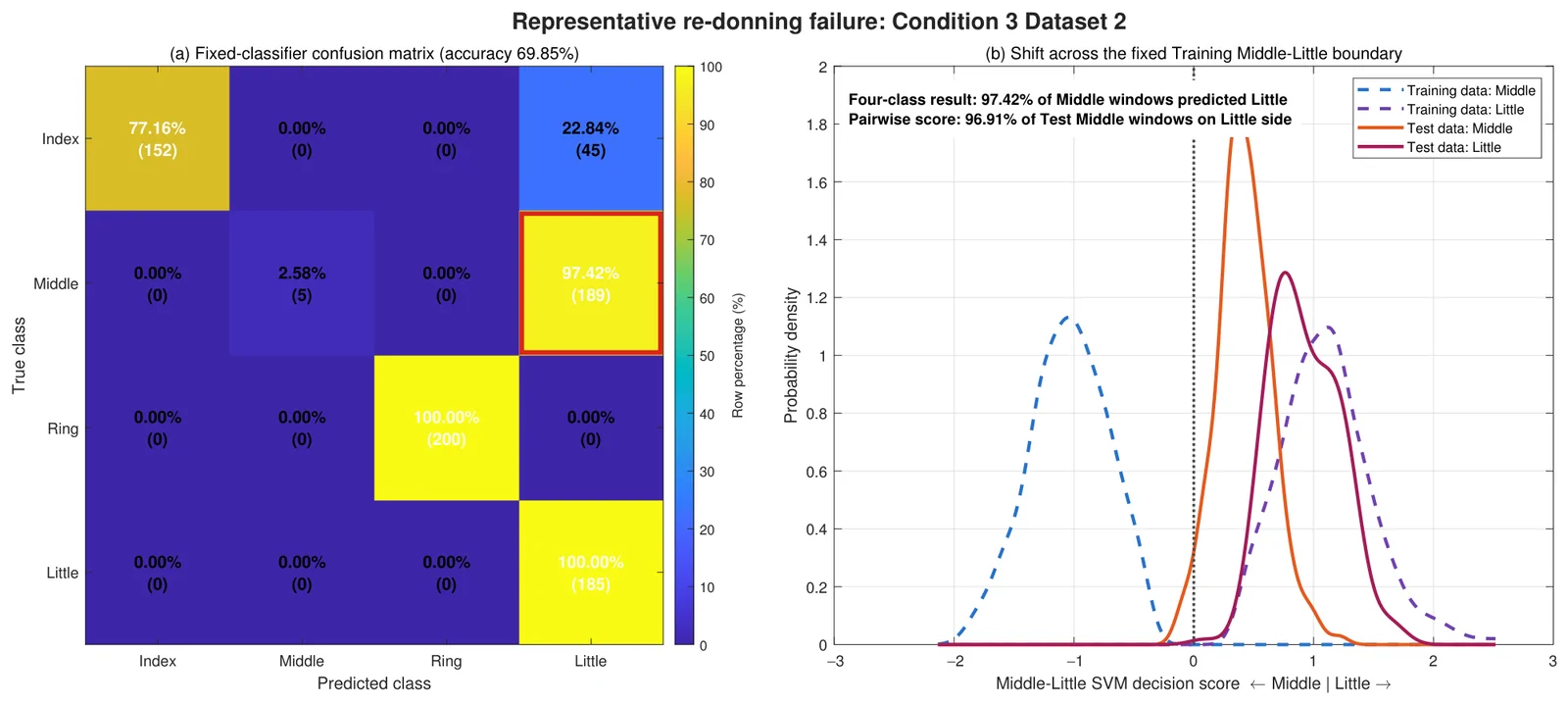
Representative class-specific failure of the fixed classifier for condition 3 Dataset 2 after sensor re-donning. (**a**) Row-normalized Stable Motion four-class ECOC-SVM confusion matrix. Values in parentheses indicate the number of windows. (**b**) Distributions of the decision scores from the Middle–Little binary learner contained in the fixed four-class ECOC classifier. Positive scores indicate the Little side of the fixed decision boundary. Of the 194 Middle windows, 189 (97.42%) were classified as Little in the four-class evaluation, and 96.91% lay on the Little side of the pairwise boundary.

Figure 4 shows condition 3 Dataset 2 as a representative case of this class-specific degradation. Of the 194 Middle windows, only 5 were correctly classified, whereas 189 (97.42%) were classified as Little. Consistently, 96.91% of the Test Middle windows were located on the Little side of the fixed Middle–Little pairwise decision boundary. The Training-data decision boundary itself remained fixed; however, sensor re-donning shifted the Middle feature distribution of the current wearing condition across this boundary, such that the boundary learned under the original wearing condition no longer separated Middle and Little appropriately. Thus, the degradation after sensor re-donning was not uniform across the four finger classes but was concentrated where the mismatch between the fixed Training-data decision boundary and the feature distribution of the current wearing condition was large.

Accordingly, the following subsection evaluates whether this condition- and class-dependent boundary mismatch could be reduced by adapting the Training data to the current wearing condition using Calibration data.

#### 3.1.3. Recovery of Stable-Motion Classification Performance by Recalibration

Table 5 compares the Stable Motion window-level four-finger accuracy of the compared conditions defined in Section 2.7. For each re-donning condition, the predictions from the two Calibration–Test directions were pooled before accuracy was calculated. The final column was calculated by pooling all 7574 eligible Stable Motion windows from the ten Test datasets.

The fixed classifier achieved a pooled accuracy of 88.90%, whereas each recalibration strategy improved the pooled result. For the same pooled Test windows, balanced accuracy and macro-F1 for the main methods are reported in the cross-participant comparison; for the Combined method these values were 98.88% and 98.87%, respectively. The illustrative offline timing summarized in Section 2.4 required 3.50 s for the first timed Combined adaptation-and-retraining repetition and 0.81±0.31 s for the subsequent 19 repetitions; these estimates exclude file input/output, label generation, construction of the fixed Training amplitude reference, MATLAB startup, Calibration acquisition, and Test prediction, and are not interpreted as a real-time bound. The two Common8 conditions increased accuracy to 96.05% for Test-side correction with the fixed classifier and 95.62% for Training-scale correction followed by retraining. The class-specific amplitude correction alone achieved 95.47%. These amplitude-only results recovered a substantial portion of the re-donning-related loss, but remained below the methods that adapted the inter-channel covariance structure.

**Figure 5:**
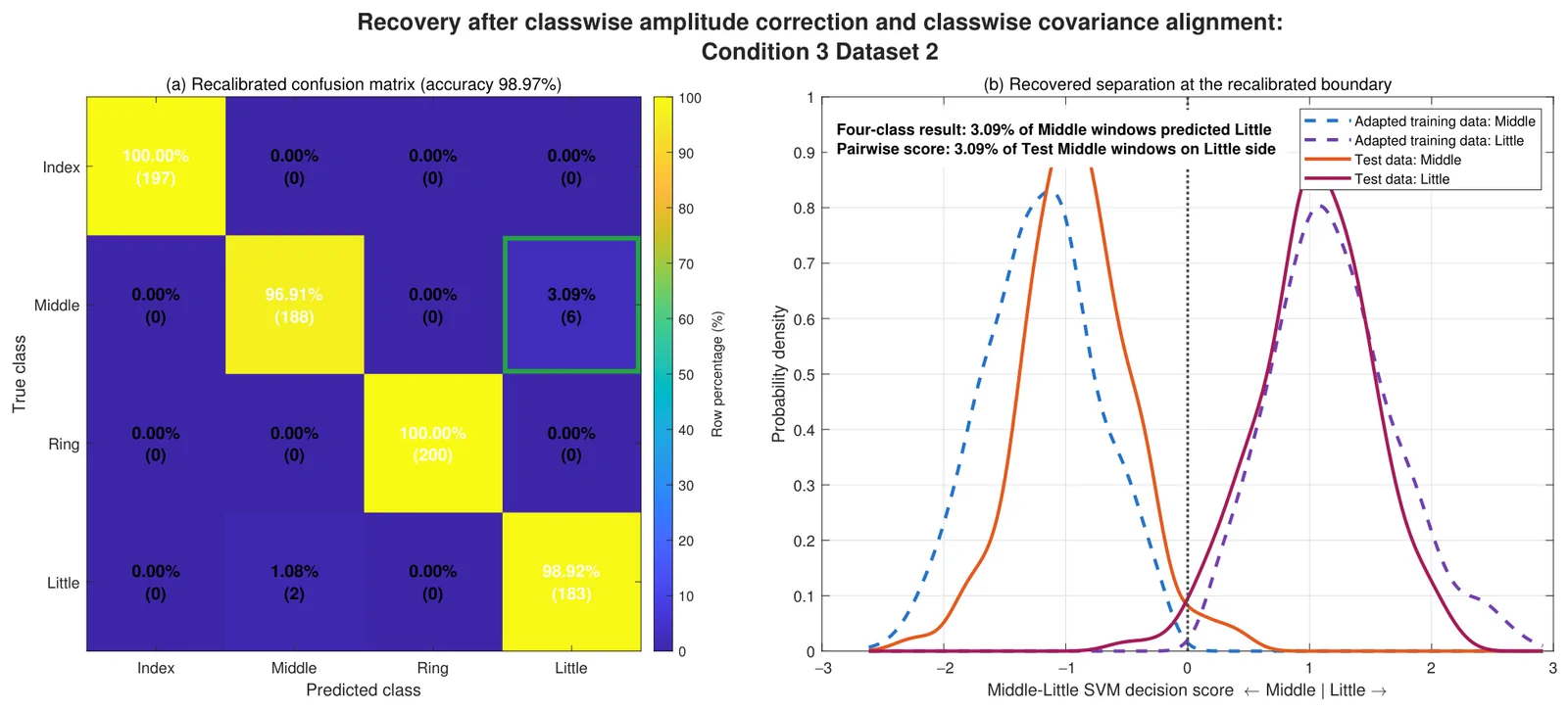
Representative recovery of class separation for condition 3 Dataset 2 after classwise amplitude correction and classwise covariance alignment. Condition 3 Dataset 1 was used as Calibration data, while Dataset 2 remained the unchanged Test dataset. (**a**) Row-normalized Stable Motion four-class ECOC-SVM confusion matrix after retraining with the adapted training data. Values in parentheses indicate the number of windows. (**b**) Distributions of the decision scores from the Middle–Little binary learner contained in the recalibrated four-class ECOC classifier. Positive scores indicate the Little side of the recalibrated boundary. Accuracy increased to 98.97%, Middle-motion recall increased to 96.91%, and Middle-to-Little errors decreased to 6 of 194 windows.

Classwise covariance alignment increased the pooled accuracy to 98.46% even without preceding amplitude correction. Applying classwise amplitude correction followed by classwise covariance alignment achieved the highest *pooled descriptive* accuracy, 98.88% (+9.98 percentage points over Fixed classifier; +0.42 over covariance-only alignment). Across the ten held-out P1 Test datasets, the Combined–Covariance increments were non-negative in all evaluations (six positive and four unchanged; mean +0.42 pp), indicating a small descriptive gain within P1 rather than a tested superiority claim. Condition-level inspection further showed that Fixed→Amplitude first-stage gains and the subsequent Amplitude→Combined increments were concentrated in conditions with marked Fixed degradation (conditions 2 and 3), whereas near-ceiling Fixed conditions changed little in the cross-participant paired analyses. The recovery was particularly pronounced in condition 3, for which accuracy increased from 68.22% to 98.25%. In contrast, condition 1 already achieved 100.00% with the fixed classifier, showing that recalibration was most consequential when re-donning produced a substantial change in the sEMG pattern. Relative to these signal-space recalibration conditions, the classical feature-space CORAL references provided complementary context. Global CORAL, used here as a standard unsupervised reference, reached 92.41% pooled accuracy. Its weaker result is consistent with estimating a single covariance from the four finger classes pooled together, thereby combining within-class and between-class structure, rather than with a claim that CORAL is intrinsically unsuitable for sEMG. The primary classical comparison is Classwise CORAL, which uses the same labeled Calibration information to align each finger class in feature space and achieved 98.06%, a level close to the proposed Classwise amplitude + Covariance result of 98.88%. To illustrate how this recovery appeared at the class-decision-boundary level, Figure 5 presents the recalibrated result for the same condition 3 Dataset 2 shown as a representative failure case in Figure 4.

In this evaluation direction, Dataset 1 from condition 3 was used as Calibration data, and Dataset 2 remained the unchanged Test dataset. After classwise amplitude correction and classwise covariance alignment, accuracy increased from 69.85% to 98.97%, and Middle-motion recall increased from 2.58% to 96.91%. The number of Middle-to-Little errors decreased from 189 to 6, and only 3.09% of the Test Middle windows remained on the Little side of the recalibrated Middle–Little pairwise boundary. Thus, retraining with the Adapted training data produced a decision boundary that was better aligned with the feature distribution of the current wearing condition and restored the separation between the Middle and Little classes.

Directly adding the labeled Calibration data to the Adapted training data did not further improve the complete method: Classwise amplitude + Covariance + Cal achieved 98.69%, compared with 98.88% without direct Calibration-data inclusion. Training from the Calibration data alone achieved 98.24%. These descriptively close results show that using the Calibration data as a reference for adapting and reusing historical Training data approached the accuracy obtained by direct current-condition retraining, without requiring the Calibration windows to be added to the final training set.

#### 3.1.4. Participant 1 Results by Calibration Repetition Count

Table 6 summarizes the Participant 1 Stable Motion window-level four-finger results obtained by varying the number of confirmed Calibration repetitions available for each finger class, following Section 2.8. Here, Calibration only denotes training the four-class ECOC-SVM directly from the eligible motion windows of the selected Calibration repetitions, without using the Training data. With only one repetition per finger, the Calibration-only classifier achieved 93.90% accuracy, whereas covariance adaptation of the Training data achieved 97.36% and the combined amplitude–covariance adaptation achieved 97.53%. Under the same one-repetition current Calibration subset, the complete Training-data-reuse pipelines therefore showed observed accuracies 3.46–3.63 percentage points higher than Calibration-only retraining; the number of classifier-training windows was intentionally not matched between these complete strategies.

Increasing the Calibration repetition count substantially improved the Calibration-only condition, from 93.90% with one repetition to 98.24% with Full Calibration. In contrast, both Training-data adaptation methods already exceeded 97% with one repetition and exceeded 98% with two repetitions. Their accuracies did not increase monotonically with every additional repetition: covariance-only adaptation was highest with three repetitions, whereas the combined procedure was highest with Full Calibration. At every evaluated repetition count, the complete Training-data-reuse pipelines showed higher observed accuracy than Calibration-only retraining, but these pipeline-level comparisons retained different amounts of historical classifier-training data.

#### 3.1.5. Supporting Signal-Level Analysis (Participant 1)

To address whether Combined adaptation reduces measurable distribution mismatch, detailed signal-level summaries were computed for Participant 1 on the ten Calibration–Test folds (Section 2.8). The covariance-distance summaries were averaged over five classes, including Rest, whereas the primary classification endpoint comprised the four finger-motion classes. Cross-participant shared-subset accuracy and adaptation-parameter stability are reported in Section 3.2.4.

**Figure 6:**
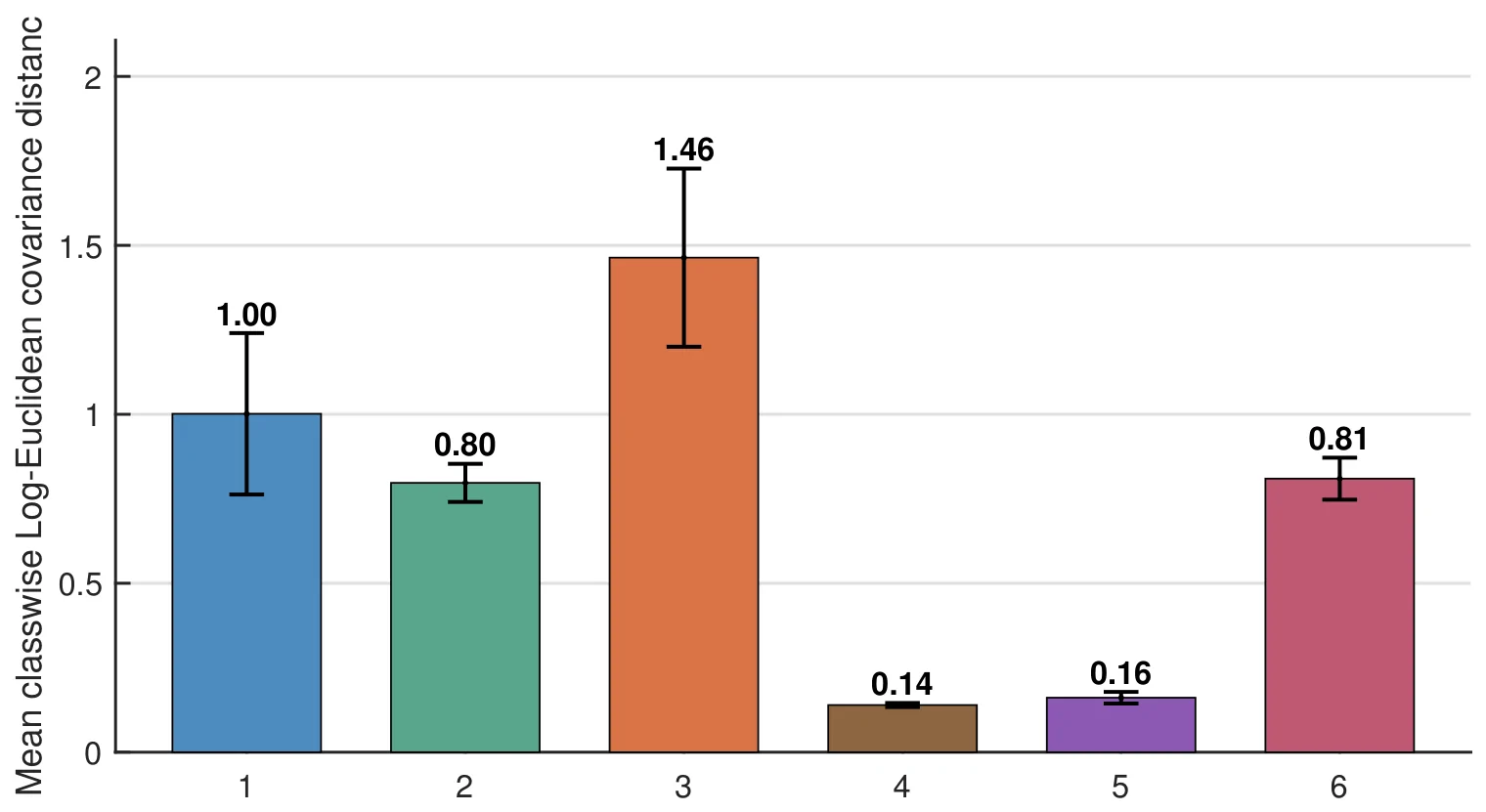
Participant 1 mean classwise log-Euclidean covariance distances across six within-wearing, pre-adaptation, and post-adaptation comparisons under full five-repetition Calibration. Bars show means and error bars show SD; condition numbers correspond to Table 7.

With full five-repetition Calibration, the mean classwise log-Euclidean covariance distances for P1 are summarized in Figure 6 and Table 7. Within-wearing reference distances were 1.00±0.24 among the five Training datasets and 0.80±0.06 between Calibration and Test data from the same re-donning condition. Before adaptation, the Training–Test distance was larger at 1.46±0.26. After Combined adaptation, the distance among adapted training datasets was 0.14±0.01, and the adapted training–Calibration distance was 0.16±0.02. These two small distances partly reflect the direct use of the Calibration covariance as the coloring target. Importantly, the Adapted training–Test distance, evaluated using Test data excluded from transformation estimation, decreased to 0.81±0.06 and was comparable to the within-wearing Calibration–Test reference of 0.80±0.06. Thus, the covariance structure of the historical Training data was moved not only toward the Calibration data used to estimate the transformation but also toward the current wearing condition represented by independent Test data.

**Figure 7:**
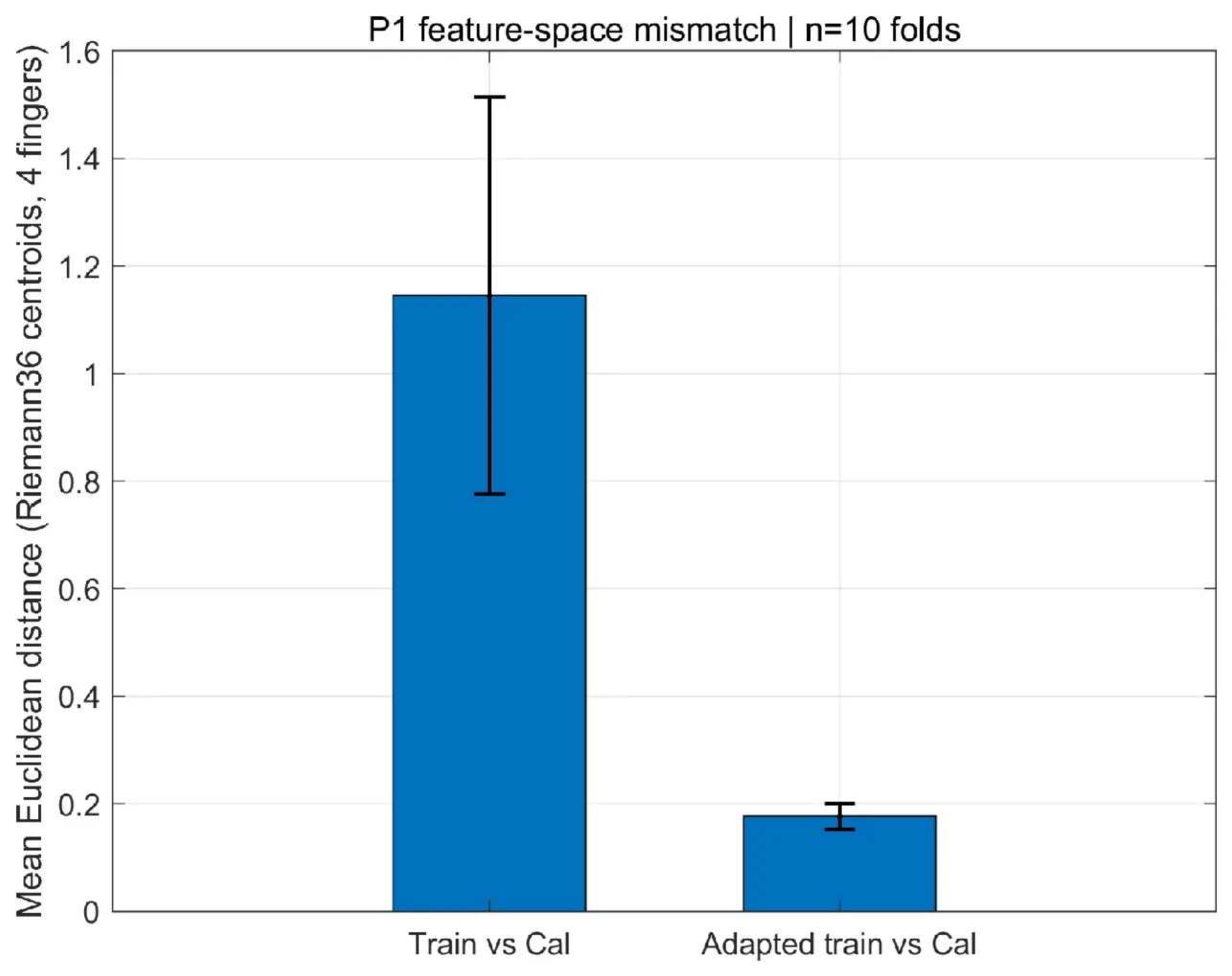
Participant 1 feature-space propagation analysis: mean Euclidean distance between classwise centroids of the 36-dimensional log-covariance features before and after Combined adaptation (Training–Calibration and Adapted training–Calibration; mean ± SD across ten folds).

**Figure 8:**
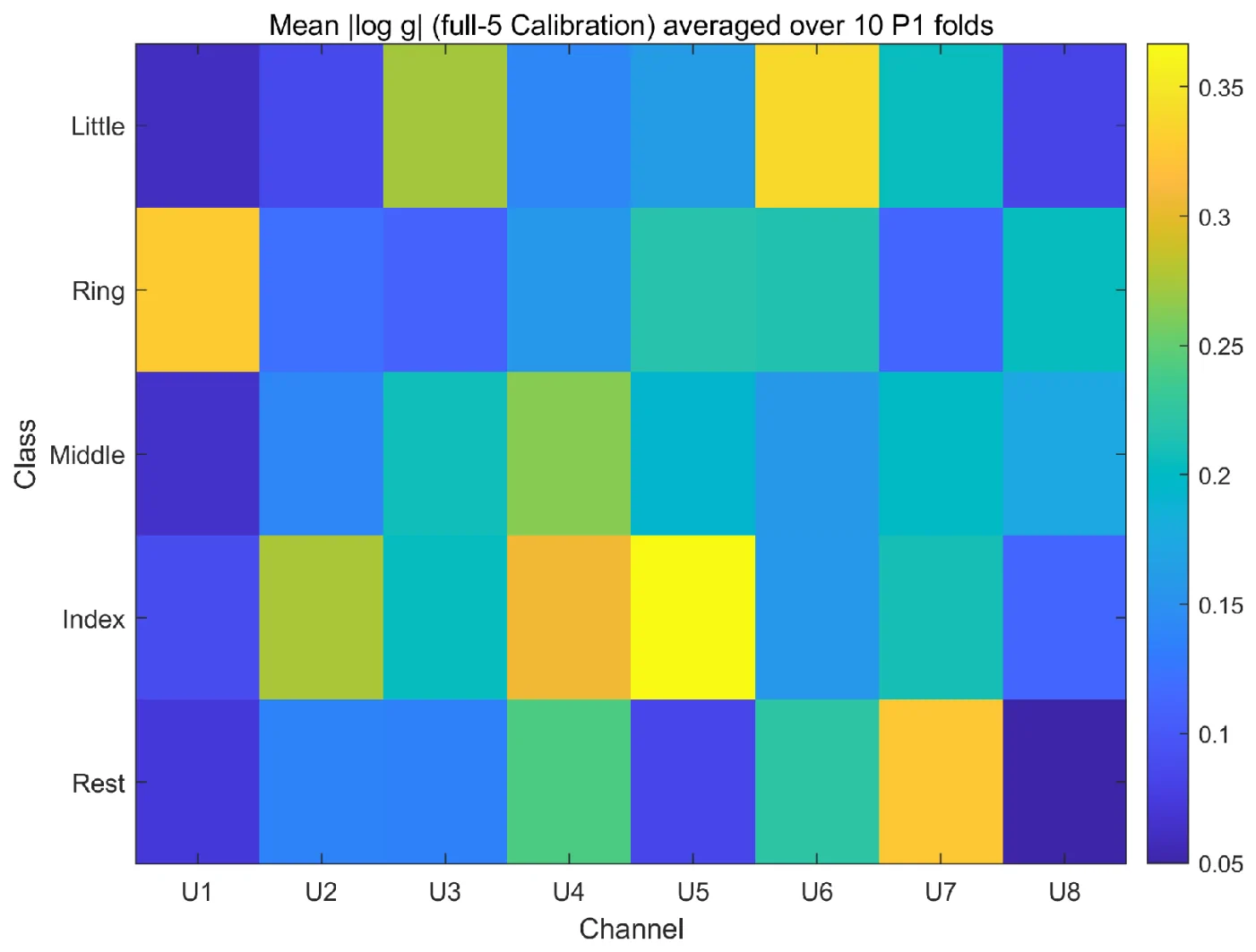
Participant 1 supporting analysis: mean absolute log-gain |logg| by class and channel for full five-repetition Calibration, averaged over the ten folds. Little denotes the little-finger class.

The corresponding mean Euclidean distance between classwise centroids of the 36-dimensional log-covariance features decreased from 1.15±0.37 (Training–Calibration) to 0.18±0.02 (Adapted training–Calibration; Figure 7). This feature-space comparison verifies that the signal-space adaptation propagated to the representation subsequently used for ECOC-SVM retraining. Because Calibration data defined the adaptation target, Figure 7 is interpreted as a propagation check rather than an independent generalization test; the independent Test-data comparison is provided by Figure 6 and Table 7. The mean absolute log-gain map summarizes channel- and class-dependent amplitude corrections across folds (Figure 8). Because each class–channel gain is the ratio of the Calibration reference amplitude to the Training reference amplitude, its absolute log magnitude directly summarizes the class- and channel-specific amplitude mismatch used by the adaptation procedure.

**Figure 9:**
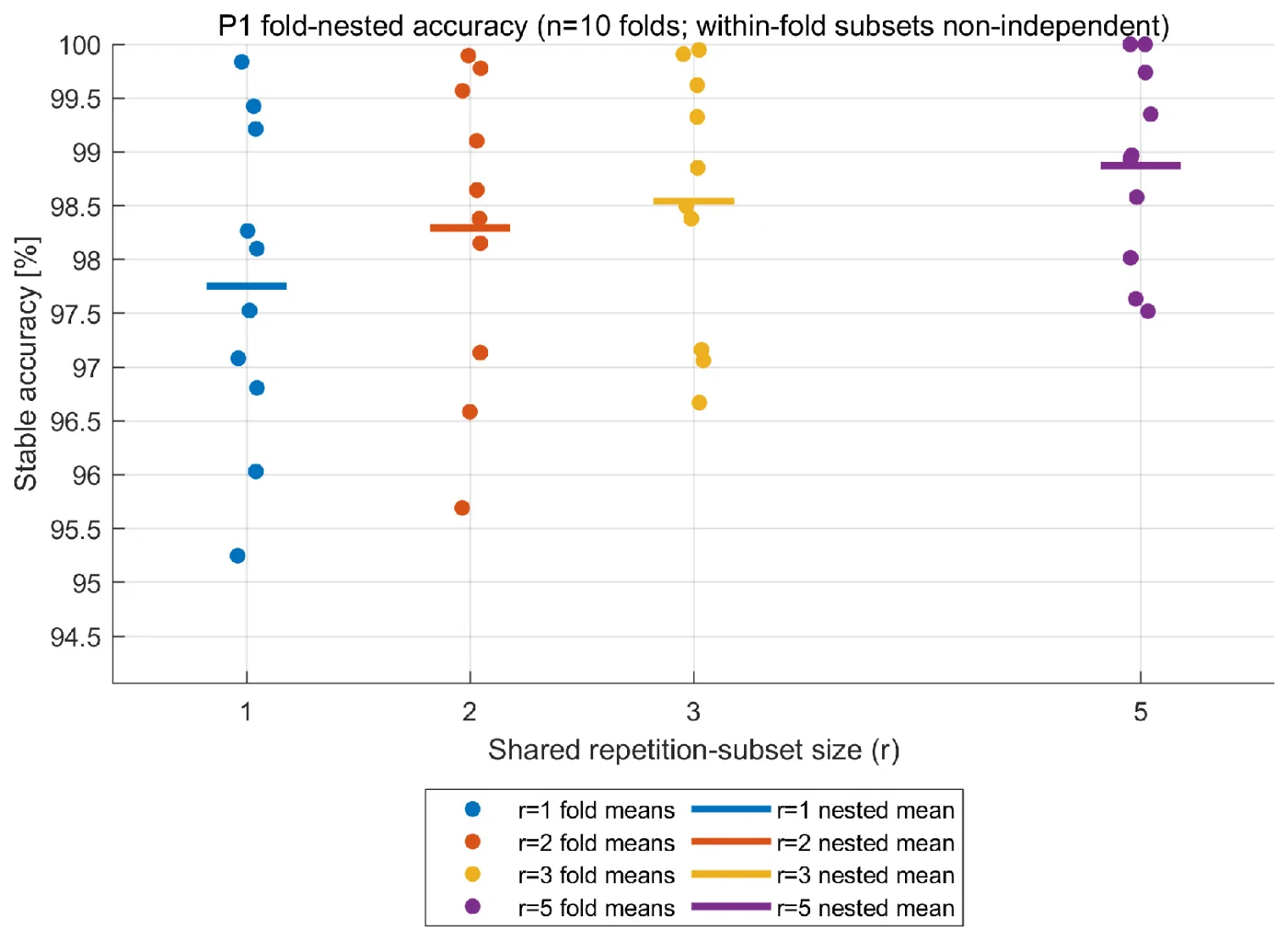
Participant 1 supporting analysis: fold-nested Combined Stable Motion accuracy for shared repetition-index subsets of size *r*. Points are within-fold subset means; horizontal marks are means across the ten folds. Within-fold subsets are not independent.

Figure 9 shows Participant 1 fold-nested Combined accuracy for shared repetition-index subsets; the corresponding P1–P3 summaries are reported in the supporting shared-subset analysis. For the P1 full-five condition, window-pooled accuracy across all 7574 Test windows was 98.88%, whereas the fold-mean was 98.87%; these two summaries are reported separately.

### 3.2. Participants 2 and 3: Three-Condition Rotating Evaluation with Six Datasets

Participants 2 and 3 were evaluated using the common motion-interval detection, labeling, and Stable Motion definition illustrated in Figure 3 and described in Section 2.2. In accordance with the predefined primary endpoint, these experiments evaluated the degradation and recovery of Stable Motion window-level four-finger classification. For each participant, all 120 commanded motion events across the six datasets satisfied the validity criteria and contributed evaluation windows.

#### 3.2.1. Same-Wearing Reproducibility and Fixed Cross-Wearing Performance

##### Participant 2: Fixed Cross-Wearing Performance

**Figure 10:**
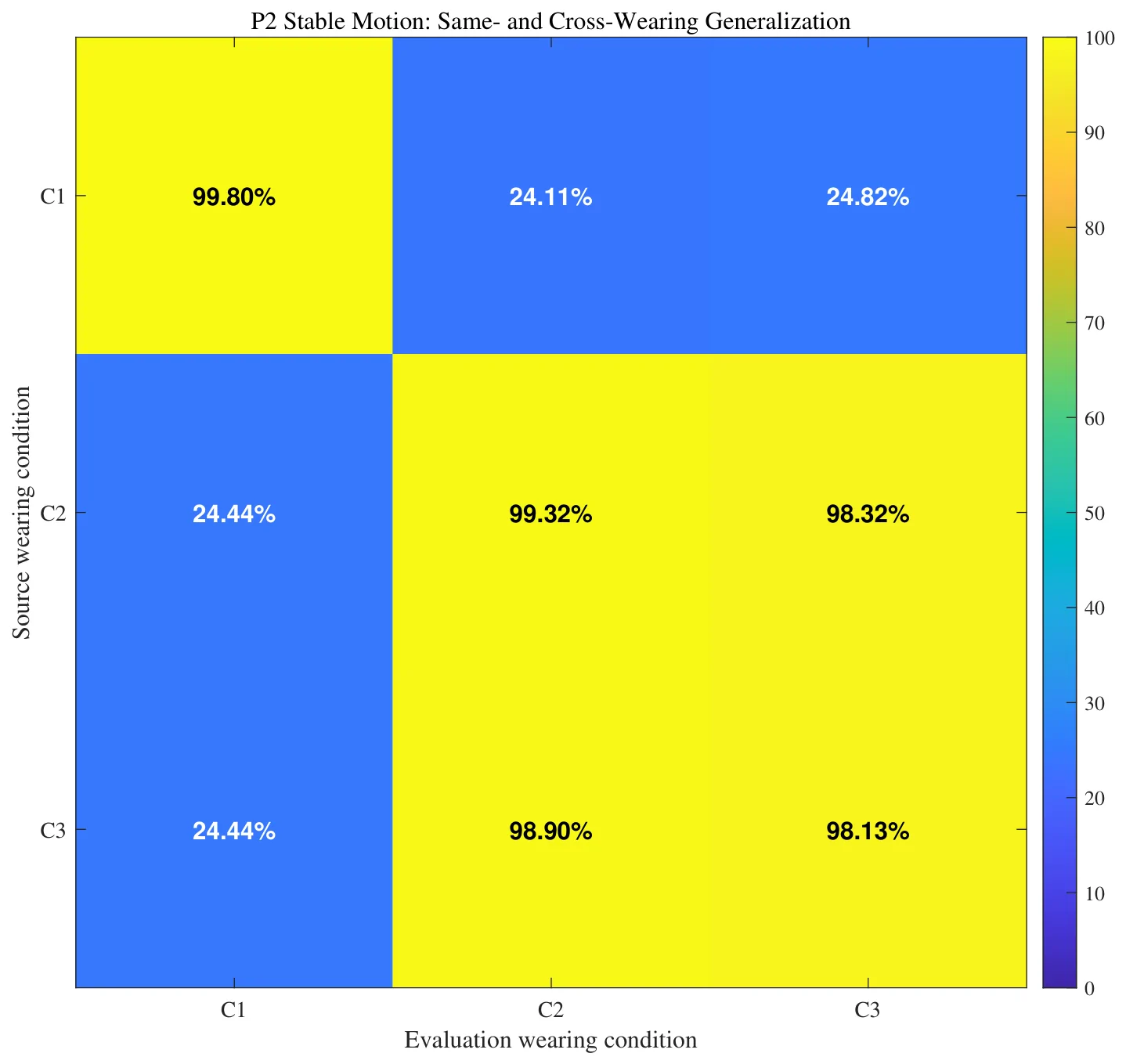
Participant 2 window-level accuracy across wearing conditions C1–C3. Diagonal cells show reciprocal same-wearing evaluation: D1 was used for training and D2 for testing, followed by reversal of these roles; the two directions were pooled. Off-diagonal cells show cross-wearing evaluation of a fixed classifier trained using both Training datasets in the row condition and tested using both datasets in the column condition. No target-wearing Calibration data were used.

Figure 10 summarizes performance before recalibration. On the diagonal, one dataset from each wearing condition was used for training, and its paired dataset was used for testing, with the two directions pooled. Accuracy remained high within the same wearing condition: 99.80% for C1, 99.32% for C2, and 98.13% for C3. Thus, the four-class classifier based on sEMG features retained finger discrimination when evaluated on an independently recorded dataset obtained without removing the device.

For the off-diagonal comparisons, the two datasets from one wearing condition were combined to form the fixed Training-data model and were evaluated without adaptation on both datasets from another wearing condition. The fixed-model accuracy decreased to 24.11–24.82% in all four directed comparisons between C1 and either C2 or C3. In contrast, C2→C3 and C3→C2 retained accuracies of 98.32% and 98.90%, respectively. These results indicate a substantial wearing mismatch between C1 and the other two conditions, whereas C2 and C3 produced mutually compatible sEMG distributions.

For a descriptive post hoc failure-case analysis, the four directed comparisons linking C1 with C2 or C3 were labeled the calibration-required pairs after inspection of the fixed-classifier results, because their accuracies were near chance. This grouping was not prespecified and was not intended to represent average performance across all six directions. The two directions between C2 and C3, for which the fixed classifier retained high accuracy, were descriptively labeled the calibration-not-required pairs. Results for all six directions are reported separately.

##### Participant 3: Fixed Cross-Wearing Performance

Participant 3 was evaluated using the same common motion-interval detection, labeling, and Stable Motion definition illustrated in Figure 3. Each of the six datasets contributed the 20 scheduled motion events (five repetitions for each of four finger classes), yielding 120 valid events in total.

Table 8 summarizes same-wearing and fixed cross-wearing performance. Reciprocal same-wearing accuracy was 95.36% for C1, 95.00% for C2, and 99.59% for C3. The lower values for C1 and C2 were direction-dependent, indicating modest dataset-specific variability even within the same wearing condition. Thus, four-class separation was high overall but was not equally reproducible for every finger and dataset.

The fixed cross-wearing classifier retained 95.02–96.57% accuracy between C1 and C2. Based on the observed fixed-classifier results, the two C1–C2 directions were assigned post hoc to the descriptive calibration-not-required subset. The four directions involving C3, for which accuracy decreased to 62.72–84.09%, were assigned post hoc to the exploratory calibration-required failure-case subset. These labels were not prespecified operational criteria.

#### 3.2.2. Recovery After Recalibration

##### Participant 2: Recalibration Recovery

Table 9 reports the six directed source-to-target wearing-condition results. The pooled calibration-required value is reported only to summarize recovery in the post hoc failure-case subset and does not replace the six-direction table. For the calibration-required pairs, the fixed classifier achieved only 24.46% accuracy. Common eight-channel scalar amplitude normalization increased accuracy to 38.37% when the classifier was kept fixed and to 37.62% after retraining, but did not resolve the mismatch. Classwise amplitude correction recovered accuracy to 96.58% in the calibration-required pool, providing the dominant first-stage recovery where the Fixed classifier had collapsed (∼24%). Across the six directed pairs, the sequential Combined–Amplitude increment remained positive but much smaller than the amplitude first-stage gain (mean +1.69 pp). Covariance-only adaptation reached 98.69% in the calibration-required pool, and Combined reached 98.97%; however, Combined did not consistently exceed covariance-only alignment across directions (Combined–Covariance: three positive, one unchanged, and two negative; cross-participant paired analysis). In the same calibration-required pool, Global CORAL remained at 42.98% and is interpreted only as an unsupervised classical reference under class-pooled feature covariance alignment. Classwise CORAL, the primary CORAL comparison, reached 99.34% and was therefore at a level comparable to the proposed Combined adaptation (98.97%). Adding the target Calibration samples produced accuracies of 99.20% for Classwise amplitude, 99.32% for covariance alignment, and 99.22% for their combination. The highest pooled accuracy among the +Cal variants was therefore obtained with covariance alignment plus Calibration, although Calibration-only retraining was similarly high at 99.25% when all five repetitions per finger were available.

**Figure 11:**
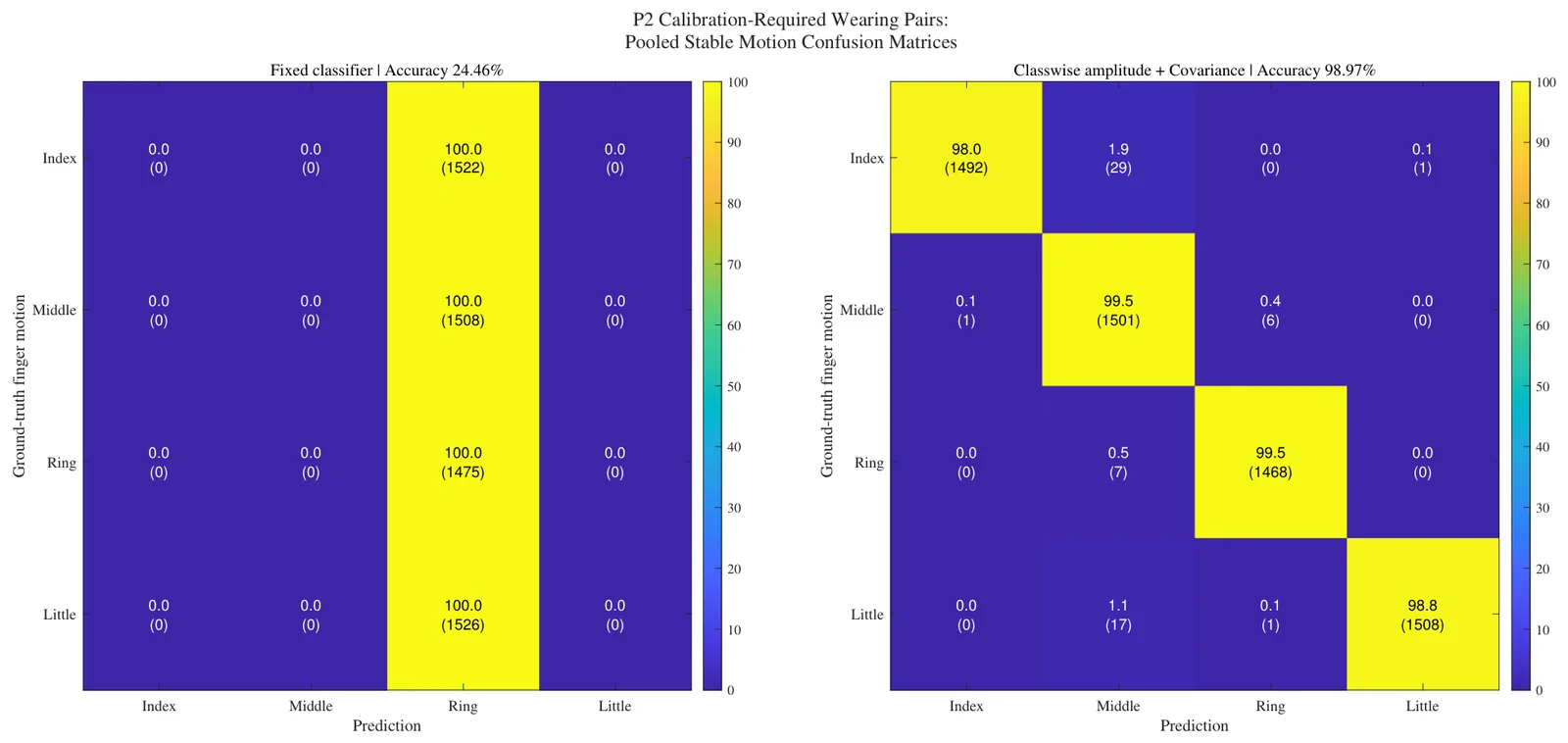
Pooled confusion matrices for the four calibration-required directed cross-wearing comparisons of Participant 2. Rows denote the commanded ground-truth finger motion and columns denote the four-class ECOC-SVM prediction. Cell colors show row-normalized percentages, with window counts in parentheses. The fixed classifier collapsed to the Ring class, whereas Classwise amplitude plus covariance adaptation restored discrimination among the four finger classes without using target Calibration windows for classifier training.

The fixed classifier in the calibration-required pairs predicted every evaluation window as the Ring class, resulting in 4556 errors among 6031 windows (Figure 11). Classwise amplitude plus covariance adaptation reduced this count to 62 errors without adding target Calibration windows to the classifier training set. The corresponding class recalls were 98.03% for Index, 99.54% for Middle, 99.53% for Ring, and 98.82% for Little. Thus, the recovery reflected restoration across all four finger classes rather than improvement in only the majority prediction. For the calibration-not-required pairs, the fixed-classifier accuracy was already 98.61%, and all evaluated strategies remained within 98.37–98.74%, showing that adaptation caused little change when the two wearing conditions were already compatible.

##### Participant 3: Recalibration Recovery

Table 10 reports all six directed source-to-target wearing-condition results; the pooled calibration-required result is an exploratory summary of the four post hoc failure-case directions. For the four calibration-required directed comparisons, the fixed classifier achieved 73.74% pooled accuracy. Common8 correction increased accuracy to approximately 85.8%, whereas Classwise amplitude and covariance alignment individually achieved 94.99% and 95.04%, respectively. Combining Classwise amplitude and covariance alignment achieved 96.72% without directly adding the target Calibration windows to classifier training, an improvement of 22.98 percentage points over the fixed classifier. Adding those windows increased accuracy to 97.71%, the highest pooled result among the compared P3 strategies. Calibration-only retraining with all five repetitions achieved a similar accuracy of 97.38%. Across all six directed pairs, classwise amplitude correction again provided substantial recovery in most directions with marked fixed degradation, whereas the sequential Combined–Amplitude increment was smaller and mixed (four positive and two negative; mean +0.76 pp). Combined adaptation was not uniformly higher than covariance-only alignment (two positive and four negative), although it provided notable additional recovery in the difficult C3→C1 and C3→C2 directions (+3.97 and +2.95 pp relative to covariance-only; cross-participant paired analysis). In the calibration-required pool, Global CORAL reached 75.61% as an unsupervised classical reference, whereas Classwise CORAL reached 97.35% and was therefore comparable to the proposed Combined adaptation (96.72%).

The pooled calibration-required accuracies for Fixed classifier, Common8 + Fixed, Common8 retrain, Classwise amplitude, Classwise amplitude + Cal, Covariance, Covariance + Cal, Classwise amplitude + Covariance, Classwise amplitude + Covariance + Cal, and Calibration only were 73.74%, 85.80%, 85.82%, 94.99%, 97.44%, 95.04%, 97.13%, 96.72%, 97.71%, and 97.38%, respectively. For the calibration-not-required C1–C2 directions, the corresponding pooled accuracies were 95.79%, 96.16%, 96.26%, 96.10%, 97.11%, 95.14%, 95.76%, 94.91%, 95.82%, and 95.18%, respectively; these methods therefore did not materially improve the already high fixed-classifier result.

The classwise recalls for Classwise amplitude plus covariance alignment were 97.39% for Index, 91.68% for Middle, 98.55% for Ring, and 99.12% for Little. After directly adding Calibration data, the corresponding recalls were 99.18%, 93.35%, 98.55%, and 99.66%. Most residual errors involved Middle windows predicted as Index, particularly in one C1 evaluation direction. The proposed recalibration therefore recovered overall four-class performance despite partial class overlap, but it did not restore all fingers to an identical level of discrimination.

#### 3.2.3. Participants 2 and 3 Results by Calibration Repetition Count

##### Participant 2: Calibration-Repetition Results

The recovery obtained from limited target data is shown in Table 11. With one target repetition per finger, Calibration-only retraining achieved 95.09% accuracy, covariance adaptation achieved 97.50%, and Classwise amplitude plus covariance adaptation achieved 98.39%. The observed accuracy of the latter Training-data-reuse pipeline was 3.30 percentage points higher than Calibration-only retraining with one repetition, 0.96 points higher with two repetitions, and 0.45 points higher with three repetitions. With all five repetitions, Calibration-only retraining reached 99.25% and was 0.28 points higher than Classwise amplitude plus covariance adaptation. Thus, the descriptive difference between the complete strategies was largest when the selected current-condition subset was smallest and narrowed as additional current repetitions were supplied; classifier-training volume was not matched between the strategies.

##### Participant 3: Calibration-Repetition Results

Table 12 shows the calibration-required results as the number of target repetitions per finger was varied. With one repetition, Classwise amplitude plus covariance adaptation achieved 95.01%, compared with 92.68% for Calibration-only retraining. With two repetitions, the combined Training-data reuse method reached 95.92%, only 0.80 percentage points below its five-repetition result. Calibration-only retraining continued to improve as additional target data were supplied and reached 97.38% with five repetitions. Participant 3 therefore showed the same descriptive pattern: the complete Training-data-reuse pipeline retained higher observed accuracy when the selected current Calibration subset was limited, whereas Calibration-only retraining improved as additional current repetitions were supplied. The classifier-training volume was not matched, and residual class overlap constrained the maximum recovery.

#### 3.2.4. Supporting Shared-Subset Robustness Analysis Across Participants

Table 13 summarizes fold-nested Combined Stable Motion accuracy from the exhaustive shared repetition-index subset analysis (Methods). Within each fold, subset outcomes were averaged before summarizing mean ± SD across folds; subsets that share repetitions were not treated as independent. P1 used ten folds; P2 and P3 used 12 folds each, covering all six directed wearing-condition pairs (not the post hoc failure-case subsets only). These fold-nested values therefore differ from the window-pooled first-*r* accuracies in the primary Calibration-amount tables for each participant.

Across the exhaustive shared-subset analysis, mean two-repetition accuracy remained within 0.18–0.58 percentage points of the corresponding full-five accuracy for all three participants. P2 showed the smallest difference, whereas P3 showed greater between-fold variability but only a 0.47-percentage-point difference in the mean. Thus, the high two-repetition performance was not restricted to the first two repetitions.

Table 14 reports deviation of subset adaptation parameters from the full-five estimates. Log-gain and Calibration covariance differences decreased as *r* increased for all participants; Participant 3 showed the largest deviations at each *r*, consistent with its greater between-fold variability in Table 13.

With full five-repetition Calibration across all six directed wearing-condition pairs for each of P2 and P3 (compatible and degraded), mean classwise log-Euclidean covariance distance decreased from 3.98±2.38 before adaptation to 0.13±0.04 after Combined adaptation for P2 and from 1.73±0.45 to 0.16±0.03 for P3; the corresponding feature-centroid distances decreased from 3.90±2.51 to 0.19±0.06 (P2) and from 1.58±0.57 to 0.25±0.05 (P3). The large P2 standard deviations reflect inclusion of all directed pairs rather than the calibration-required subset alone.

### 3.3. Cross-Participant Descriptive Comparisons

Table 15 summarizes paired method differences; the subsequent tables summarize evaluation-unit variability, complementary classification metrics, and condition- and direction-level contribution patterns across P1–P3 after the participant-specific results have been presented. These summaries are descriptive within-participant comparisons rather than population-level significance tests.

Table 16 shows that Fixed-classifier performance varied widely across evaluation units, whereas Amplitude, Covariance, and Combined recalibration concentrated the unit-level accuracies in a narrower high range. The corresponding paired differences (Table 17) indicate that the Amplitude–Fixed recovery was large on average for all three participants, while the additional Combined–Covariance increment remained small. For P2 and P3, the evaluation-unit bootstrap interval for Combined–Covariance included zero, so this small sequential increment should not be interpreted as a stable unit-level advantage over covariance-only adaptation.

Table 18 shows that balanced accuracy and macro-F1 closely tracked overall accuracy for the recovered conditions, whereas the P2 Fixed collapse produced a much lower macro-F1 than accuracy because errors were concentrated in class-asymmetric confusions. Together with Table 16, these summaries indicate that method comparisons should not rely on a single pooled accuracy value alone.

Using the already reported Stable Motion accuracies, Table 19 reports first-stage amplitude recovery (ΔAmp) and the sequential increment obtained by subsequently applying classwise covariance alignment (Δ(Comb–Amp)). Across participants, large ΔAmp values occurred primarily when Fixed-classifier transfer was difficult (for example, P1 C3 and the P2 transfers near 24% Fixed accuracy). The sequential covariance increment was smaller on average (P1 +3.37 pp, P2 +1.69 pp, and P3 +0.76 pp at the paired-evaluation level in Table 15) and varied across conditions and directions; near-ceiling Fixed evaluations showed little change. These accuracy deltas do not directly quantify amplitude or covariance distribution offsets. Because electrode translation/rotation was not recorded, Fixed-classifier degradation is used only as a descriptive indicator of cross-wearing transfer difficulty, not as a direct measure of distribution-mismatch severity or a physical displacement dose–response.

### 3.4. Supporting Analysis: Fixed-Model Correction Without Classifier Retraining

The participant-specific analyses in Section 3.1 and Section 3.2 addressed Training-data adaptation and Calibration repetition count. An additional supporting analysis examined whether a simpler recalibration strategy could recover performance by correcting only the incoming Test signals toward the Training-data condition while retaining the fixed classifier. The detailed comparison below is reported for Participant 1; the corresponding Common8 + Fixed results for Participants 2 and 3 are given in Table 9 and Table 10. Table 20 presents the Participant 1 results.

Among the fixed-model conditions, Common8 amplitude correction achieved the highest pooled accuracy, 96.05%, reducing the number of errors from 841 to 299. Global covariance correction improved accuracy relative to Fixed classifier but reached only 93.69%. Replacing the second full covariance alignment with a correlation-only alignment after Common8 produced 93.11%. Thus, preserving the Common8-corrected channel variances while aligning the pooled inter-channel correlation structure did not improve the simpler Common8 correction. Common8 exceeded the correlation-aligned condition in all conditions except condition 4.

The class-specific recalls further showed why pooled covariance matching was insufficient. With GlobalCov + Fixed, Middle-motion recall increased to 98.89%, but Little-motion recall decreased to 77.71%, with 391 Little-to-Middle errors. The combined Common8 and global correlation condition showed the same pattern: Middle-motion recall was 99.00%, whereas Little-motion recall was 77.10%, with 406 Little-to-Middle errors. Therefore, even when the Common8-corrected channel variances were retained, matching one pooled Motion correlation structure did not preserve the class-specific relationship between the Middle and Little distributions.

These results indicate that Common8 amplitude correction provides a computationally simpler option when classifier retraining is undesirable. Nevertheless, its pooled accuracy remained 2.83 percentage points below the 98.88% obtained by the complete Classwise amplitude + Covariance Training-data adaptation procedure. The class-specific adaptation and retraining procedure provided the highest pooled descriptive accuracy in this comparison.

## 4. Discussion

The results from Participants 1–3 show how distribution mismatch associated with sensor re-donning affected Stable Motion four-finger classification, how Training-data adaptation using limited Calibration data recovered performance, and how the number of Calibration repetitions per finger affected the observed accuracy relationship between complete Training-data-reuse and Calibration-only retraining strategies. All three participants were evaluated using the same motion-interval definition and four-class ECOC-SVM endpoint.

### 4.1. Baseline Finger Discrimination and Performance Degradation After Sensor Re-Donning

For Participant 1, the within-Training Stable Motion window-level four-finger accuracy of 99.76% indicates that the four finger classes were highly separable across repeated recordings while the sensor-wearing condition remained unchanged. In contrast, the fixed classifier achieved a pooled accuracy of 88.90% after sensor re-donning. The magnitude of this reduction depended strongly on the resulting wearing condition: condition 1 retained 100.00% accuracy, whereas condition 3 achieved only 68.22%. This variability is consistent with previous reports that the effect of electrode displacement depends on the displacement condition [3]. The magnitude of degradation likewise depended strongly on the resulting wearing condition in the present study, ranging from near-complete preservation to substantial loss of classification accuracy. Because the protocol reproduced naturally occurring re-donning conditions, these results are interpreted at the wearing-condition level rather than as a physical displacement dose–response. The present findings further indicate that substantial recalibration may not be necessary when sensor re-donning does not produce a large shift in the feature distribution of the current wearing condition relative to the Training data used to train the original classifier.

The Participant 2 results reproduced this condition-dependent behavior under the three-condition rotating evaluation. Same-wearing accuracy remained between 98.13% and 99.80% across the three wearing conditions, demonstrating that the sEMG-based classifier was reproducible across independently recorded datasets when the device was not removed. Fixed cross-wearing performance, however, depended strongly on the pair of wearing conditions. Accuracy remained 98.32–98.90% between C2 and C3, but decreased to 24.11–24.82% for all directed comparisons linking C1 with either of those conditions. Together with the Participant 1 results, this finding indicates that re-donning does not inevitably invalidate a previous classifier. Poor transfer was observed when the new wearing condition differed substantially from the Training-data feature distribution, consistent with a large distribution mismatch.

Participant 3 further showed that same-wearing reproducibility itself need not be uniform across datasets or fingers. Reciprocal same-wearing accuracy was 95.36%, 95.00%, and 99.59% for C1, C2, and C3, respectively. The lower results were direction-dependent, indicating modest dataset-specific variability even within the same wearing condition. Fixed transfer remained 95.02–96.57% between C1 and C2, but decreased to 62.72–84.09% in the four directions involving C3. Therefore, individual physiology, sensor location, and repetition variability may produce partial overlap among four finger classes even before the additional mismatch caused by re-donning is considered.

For Participant 1, the degradation was also concentrated in particular finger classes. Ring motion remained correctly classified in all ten fixed-model Test datasets, whereas Middle and Index recall decreased markedly in conditions 2 and 3. In the representative condition 3 Dataset 2 case, 189 of 194 Middle windows were classified as Little. As shown in Figure 4, 96.91% of the Middle windows were located on the Little side of the fixed Middle–Little decision boundary. This pattern suggests that re-donning did not simply increase nonspecific classification noise. Rather, it changed the correspondence between the feature distribution of the current wearing condition and the decision boundary learned from the original Training data. Because electrode displacement can alter relative channel amplitudes and spatial relationships among channels [4,6], the resulting mismatch can be expected to differ across motions whose muscle-activation patterns emphasize different sensor locations. The class-dependent errors observed here therefore support the use of recalibration procedures that preserve class-specific information rather than applying only a single global correction.

Participant 2 exhibited a more severe form of mismatch in the four calibration-required comparisons: the fixed classifier predicted every Stable Motion window as Ring. This collapse differs from the more localized class confusions observed for Participant 1, but it leads to the same interpretation. Re-donning can alter the mapping between class-specific sEMG distributions and previously learned decision regions, and the form and severity of that alteration depend on the resulting wearing condition.

### 4.2. Recovery of Finger Discrimination Through Personalized Recalibration

Classwise amplitude correction provided the most consistent first-stage recovery across participants when substantial cross-wearing degradation was present, while little benefit was observed where the Fixed classifier already retained near-ceiling accuracy (Table 19). For Participant 1, amplitude correction increased pooled Stable Motion accuracy from 88.90% to 95.47%. This improvement supports the view that changes in the relative amplitude characteristics of the eight channels were an important component of the cross-wearing mismatch, consistent with channel-amplitude normalization under electrode displacement [4]. However, amplitude correction alone did not recover the performance obtained by the covariance-based methods in P1, indicating that the mismatch was not limited to independent changes in channel magnitude. Covariance alignment after amplitude correction provided a smaller and more condition-dependent sequential increment than the first-stage amplitude recovery (Table 15, Table 17 and Table 19). Mean Combined–Amplitude differences were approximately +3.37 pp for P1, +1.69 pp for P2, and +0.76 pp for P3 at the paired-evaluation level. Evaluation-unit bootstrap intervals for these mean differences excluded zero for P1 and P2 but included zero for P3, consistent with direction-dependent increments for P3 (Table 17). For P1, combining amplitude and covariance adaptation achieved the highest pooled descriptive accuracy (98.88%; +0.42 pp over covariance-only), with non-negative Combined–Covariance differences in all ten Test evaluations and an evaluation-unit bootstrap interval of [0.18,0.67] pp. The corresponding increments were less consistent across P2/P3 directions, where the bootstrap intervals included zero. These results characterize contribution patterns across wearing conditions rather than physical electrode-displacement magnitudes and are interpreted as within-participant descriptive evidence. Multi-channel covariance representations capture relationships among electrode signals that are not retained by treating the channels independently [6,7]. In the proposed procedure, these relationships were aligned separately for each finger class, allowing the inter-channel structure of each finger class in the Training data to be aligned toward the corresponding current Calibration distribution. The P1 covariance-distance analysis provides signal-level support for this interpretation (Figure 6 and Table 7). Before adaptation, the Training–Test distance exceeded both within-wearing reference distances. After Combined adaptation, the Adapted training–Test distance was nearly identical to the within-wearing Calibration–Test reference, even though Test data were not used to estimate the transformation. The still smaller distances among Adapted training datasets and between Adapted training and Calibration should be interpreted in light of the direct use of the Calibration covariance as the coloring target. Thus, the independent Test comparison, rather than the Adapted training–Calibration distance alone, provides the stronger evidence that the historical signals were moved toward the current wearing condition.

The recovery observed in the most strongly affected dataset illustrates the observed effect of matching the training distribution to the current wearing condition. In the representative condition 3 Dataset 2 case, the complete procedure increased Middle-motion recall from 2.58% to 96.91% and reduced Middle-to-Little errors from 189 to 6. This recovery is consistent with a decision boundary learned from the Adapted training data being better matched to the class distributions of the current wearing condition.

Participant 2 showed a similar recovery pattern. In the four calibration-required directed comparisons, the fixed classifier achieved 24.46% accuracy. Classwise amplitude correction increased accuracy to 96.58%, covariance alignment increased it to 98.69%, and their combination reached 98.97% without adding target Calibration windows to classifier training. The combined transformation reduced the number of errors from 4556 to 62 and restored recall to 98.03–99.54% across all four finger classes. Thus, amplitude and covariance adaptation recovered not merely the dominant prediction but the complete four-class structure. When target Calibration samples were also included, covariance alignment plus Calibration produced the highest pooled accuracy of 99.32%.

Participant 3 extended this finding to a case with greater within-wearing class overlap. Across the four calibration-required comparisons, Classwise amplitude + Covariance adaptation increased accuracy from 73.74% to 96.72%, and direct inclusion of Calibration windows increased it to 97.71%. The transformation restored Index, Ring, and Little recall to 97.39–99.12% without direct Calibration-window inclusion, although Middle recall remained lower at 91.68%. This result indicates that the proposed approach can recover most of the re-donning-related loss even when some class overlap and within-wearing variability are already present.

The present study also includes classical feature-space CORAL [16] as an external reference: Global CORAL is reported as a standard unsupervised baseline under class-pooled covariance alignment, whereas Classwise CORAL is treated as the primary classical comparison because the protocol is multi-class and labeled Calibration data are already available.

The full-repetition Calibration-only results provide a useful reference for interpreting these recoveries. Calibration-only classifiers were trained directly on data from the current wearing condition and achieved 98.24%, 99.25%, and 97.38% for P1–P3, respectively. By contrast, Combined adaptation used the current Calibration data to transform historical Training data acquired under a different wearing condition and achieved 98.88%, 98.97%, and 96.72%, respectively. These descriptively close accuracies indicate that recalibrated historical Training data approached the performance obtained by constructing a classifier directly from current-wearing-condition data within the evaluated participants and conditions. The comparison is interpreted descriptively because the classifier-training pools differed between the two strategies.

### 4.3. Exploratory Performance with Retrospectively Reduced Calibration Data

The exploratory retrospective calibration-amount analysis compared complete strategies under the same selected current Calibration repetitions but with intentionally different classifier-training pools: Calibration-only used the selected current-condition windows directly, whereas Training-data reuse retained the historical Training recordings after adaptation. For Participant 1, with one confirmed repetition per finger, Calibration-only training achieved 93.90% accuracy, whereas covariance-adapted Training data and combined amplitude–covariance Adapted training data achieved 97.36% and 97.53%, respectively. Both Training-data adaptation methods exceeded 98% with two repetitions per class. Their observed accuracies were higher than Calibration-only at every evaluated Calibration amount for P1, with the largest difference when the selected current subset was smallest. These results compare the complete data-reuse and current-data-only strategies under the same selected current-Calibration burden, with the historical Training pool retained only by the data-reuse strategy.

Training data contain repeated examples of the participant’s motion patterns and therefore provide substantially more training observations than a small Calibration dataset. The proposed method retains this Training-data information while altering its amplitude and covariance characteristics using the current Calibration dataset as a target. This differs from discarding the Training data and rebuilding the classifier from the Calibration data alone. Previous recalibration studies have combined prior-session information with limited current-session data by adapting classifier parameters, updating decision boundaries, or transforming current-session representations [9,10,11,12].

The present results support an alternative strategy in which the user-specific Training signals themselves are adapted toward the current wearing condition before feature extraction and classifier retraining.

For Participant 1, directly adding the Calibration samples to the Adapted training data did not improve the complete method: Classwise amplitude + Covariance + Cal achieved 98.69%, compared with 98.88% without direct inclusion. This result does not imply that Calibration samples are generally uninformative, because they were essential for estimating the adaptation parameters. Instead, it indicates that their principal value in the present setting was as a reference distribution for adapting the larger Training dataset. The primary three-participant Calibration-amount comparison used the first *r* valid repetitions in temporal order. Supporting exhaustive shared-subset analyses for P1–P3 showed that fold-nested mean two-repetition combined accuracy remained within 0.18–0.58 percentage points of the corresponding full-five accuracy (Table 13) and that deviations from the full-five adaptation parameters decreased as *r* increased (Table 14; Participant 1 subset accuracies in Figure 9). These results reduce concern that limited-data calibration performance depended only on the earliest repetitions and support the feasibility of retaining historical Training information when only a limited current labeled subset is available.

Participants 2 and 3 showed the same qualitative limited-data pattern. For Participant 2, the observed Classwise amplitude + Covariance minus Calibration-only accuracy difference decreased from 3.30 percentage points with one repetition to 0.96 and 0.45 points with two and three repetitions, respectively; with all five repetitions, Calibration-only retraining reached 99.25% and was 0.28 points higher than the combined adaptation result of 98.97%. For Participant 3, the combined method achieved 95.01% versus 92.68% for Calibration-only retraining with one repetition and reached 95.92% with two repetitions, whereas Calibration-only retraining continued to improve to 97.38% with five repetitions. This pattern supports Training-data reuse particularly when current labeled data are scarce, while Calibration-only retraining becomes increasingly competitive as more current repetitions are available.

Taken together, the three-participant results provide preliminary evidence for evaluating a candidate acquisition strategy in which participant-specific Training data are initially acquired across multiple repetitions and subsequently adapted to each new sensor-wearing condition using a small labeled Calibration set, with the classifier retrained using the resulting Adapted training data. With two Calibration repetitions per finger, Classwise amplitude + Covariance Training-data adaptation achieved 98.65% for Participant 1, 99.10% for Participant 2, and 95.92% for Participant 3. The corresponding Calibration-only classifiers trained from all five repetitions per finger achieved 98.24%, 99.25%, and 97.38%, respectively. This practical juxtaposition compares complete acquisition strategies with different Calibration amounts and classifier-training pools. Under the acquisition timing used in this study, the five-repetition protocol required approximately 159 s: 10 s of initial Rest, four finger blocks each containing 3 s of preparation, 3 s of countdown, and five 3-s motion/3-s return-to-Rest pairs, followed by 5 s of final Rest. If each finger block were reduced to two repetitions while retaining all fixed preparation and Rest periods, the theoretical recording duration would be 10+4[3+3+2(3+3)]+5=87 s. This corresponds to a reduction of approximately 72 s, or 45%, relative to the present protocol. This 45% estimate is a practical implication of the retrospective analysis; a prospectively shortened two-repetition acquisition protocol remains to be evaluated.

The effect of directly adding current Calibration samples was also not identical across participants. It did not improve the complete method for Participant 1, whereas for Participant 2 it increased covariance-based accuracy from 98.69% to 99.32% and Classwise amplitude + Covariance accuracy from 98.97% to 99.22%. For Participant 3, direct inclusion increased the combined result from 96.72% to 97.71%. These differences suggest that the potential role of Calibration samples—adaptation reference, direct training data, or both—may depend on the participant and the severity of the wearing mismatch.

The post hoc descriptive contrast between the calibration-required and calibration-not-required pairs suggests a hypothesis for prospective evaluation. For Participant 2, the fixed classifier already achieved 98.61% for the mutually compatible C2 and C3 conditions, and adaptation changed pooled accuracy only within 98.37–98.74%. Recalibration provided little benefit in this observed case, whereas the C1-linked comparisons showed substantial benefit from adaptation. For Participant 3, the compatible C1–C2 comparisons retained 95.79% pooled accuracy with the fixed classifier, whereas the four comparisons involving C3 averaged 73.74% before recalibration. On those compatible pairs, Classwise amplitude + Covariance adaptation reached 94.91% and therefore did not improve the already high fixed-classifier result. These post hoc observations suggest that a compatibility check between the current wearing distribution and the stored model may help determine when recalibration is beneficial and provide a hypothesis for prospective development and validation of a decision rule.

### 4.4. Fixed-Model Correction Without Classifier Retraining

The complete P1 Classwise amplitude + Covariance procedure provided the highest *pooled descriptive* Stable Motion accuracy among the compared Training-data adaptation strategies, but required access to the Training signals, feature re-extraction, and retraining of the four-class ECOC-SVM. Common8 Test-side correction offered a simpler alternative: it retained the fixed classifier and increased accuracy to 96.05% by applying only class-independent channel gains to incoming Test signals. This option may be useful when retaining or reprocessing previously acquired Training data is impractical or when classifier retraining is undesirable. Its accuracy, however, remained 2.83 percentage points below that of the complete method.

The other fixed-model results show why the simpler transformation should not be made more complex without preserving class information. A single pooled Motion covariance correction improved accuracy from 88.90% to 93.69%, but it increased Little-to-Middle errors, and correlation alignment after Common8 achieved 93.11% and therefore did not outperform Common8 alone. Matching one pooled inter-channel structure could improve one class while moving another class in an unfavorable direction. By contrast, the complete recalibration estimated separate transformations for Rest and each finger class and retrained the decision boundaries using the Adapted training data.

The choice between these approaches therefore depends on the intended use case. Common8 correction reduces the recalibration procedure to estimating and applying eight channel gains and does not require test-time class labels. The complete method requires labeled Calibration data and offline reconstruction of the participant-specific classifier, but its class-specific transformations better preserve the relationships among the finger distributions. The poor performance of pooled covariance and correlation corrections relative to Common8 also shows that adding a more complex global transformation does not necessarily provide a better fixed-model solution. These results suggest that additional transformation complexity was advantageous in the present setting when class-specific information was available during recalibration, and the classifier was subsequently retrained; ground-truth class information remains unnecessary during actual testing.

The Participant 2 calibration-required results further delimit the role of the simplified fixed-model option. Common8 Test-side correction increased accuracy from 24.46% to only 38.37%, whereas Classwise amplitude + Covariance adaptation and retraining reached 98.97%. Thus, a class-independent gain correction may be a useful low-cost option for moderate mismatch, as observed for Participant 1, but it was insufficient when cross-wearing mismatch caused complete class collapse. The computationally simpler option should therefore be considered a conditional alternative rather than a replacement for classwise adaptation.

Participant 3 showed an intermediate mismatch: Common8 Test-side correction increased accuracy from 73.74% to 85.80%, whereas Classwise amplitude + Covariance adaptation and retraining achieved 96.72%. Thus, scalar gain correction provided partial recovery but again did not replace classwise adaptation.

Together, these findings support the feasibility of class-specific Training-data adaptation across the evaluated participants and sensor-wearing conditions.

## 5. Limitations

This study involved three participants and used a within-participant design in which an initial participant-specific model was constructed and then evaluated after sensor re-donning. The analysis therefore demonstrates the feasibility of recovering Stable Motion finger discrimination within the recorded participants and wearing conditions. The evaluation windows were repeated, overlapping observations within participants rather than independent population samples, and the reported window-level accuracies should not be interpreted as population-level performance estimates. In particular, because consecutive windows overlapped by 80%, the number of Stable Motion windows was not used as an independent sample size for confidence intervals or hypothesis tests. Uncertainty was instead summarized at the dataset or directed-pair level (Table 16 and Table 17). Because the directed pairs reuse recordings across source and target assignments and the number of evaluation units was small, these bootstrap intervals were interpreted as descriptive within-participant uncertainty summaries rather than population-level inferential intervals. Further validation is required in a larger and more diverse cohort spanning sex, age, forearm anatomy, and intended end-user populations, including individuals with upper-limb absence or impaired finger movement, whose residual muscle anatomy, motor strategies, and sEMG distributions may differ from those of anatomically intact participants.

The P1 and P2/P3 data configurations were designed to address the same degradation-and-recovery question but did not constitute a controlled comparison of Training-data volume. Consequently, the P2/P3 results obtained with two initial Training datasets do not establish that two datasets are equivalent to the five Training datasets used for P1.

The separability of the four finger classes was not uniform across participants or wearing conditions. Participant 3 showed dataset- and direction-dependent variability even within nominally unchanged wearing conditions. The proposed method recovered most of the subsequent cross-wearing loss, but Middle recall remained lower than that of the other classes. Recalibration can reduce a distribution mismatch between Training data and the current wearing condition, but it cannot guarantee complete separation when the underlying class distributions already overlap because of individual physiology, sensor position, or motion variability. The P3 result should therefore be interpreted as evidence of recovery under partial class overlap, not as evidence that recalibration makes all four classes perfectly separable.

The low-density sEMG sensor used in this study may have limitations in classifying individual finger motions depending on its placement on the forearm. The present study intentionally focused on naturally occurring wearing-condition changes after complete sensor removal and re-donning rather than on controlled electrode-shift magnitudes. However, because neither the initial sensor position nor the displacement after re-donning was quantitatively recorded, the present study cannot establish a quantitative relationship among physical electrode displacement, distribution mismatch, classification degradation, and recalibration benefit. Future studies should quantify the initial sensor placement and the rotation and translation caused by re-donning, and analyze their relationships with classwise feature distributions, classification errors, and recalibration performance.

The fixed movement order was used to standardize the Training and Calibration protocol across participants, datasets, and wearing conditions and to associate detected motion intervals with the intended finger commands because the sEMG stream did not independently encode finger identity. However, because the same order was also used in the Test recordings, finger identity was confounded with temporal position within each recording. Accordingly, the present results evaluate recalibration under the standardized isolated-motion protocol and do not establish order-independent finger classification or continuous-control performance. The choice of five repetitions per finger was not established as optimal, and independence from the fixed movement order was not evaluated. Future experiments may retain a controlled calibration procedure while evaluating independent Test recordings with randomized or counterbalanced finger orders.

The Calibration-repetition analysis directly evaluated whether reusing prior Training data could maintain high classification performance when only limited current Calibration data were available. The primary comparison used the first *r* valid repetitions in temporal order from full five-repetition recordings. Supporting exhaustive shared-subset analyses were additionally performed for P1–P3 using the same recordings; prospectively shortened Calibration recordings were not acquired or evaluated. The exhaustive subset analysis addressed sensitivity to repetition selection, but it did not evaluate independently randomized or counterbalanced Calibration acquisition orders.

The evaluation was deliberately restricted to Stable Motion, where the finger-specific sEMG information within each motion was sufficiently established for the four fingers to be most reliably distinguishable. This design examined whether classification deteriorated after sensor re-donning even in this information-rich interval and whether recalibration restored it. If performance cannot be maintained in this stable interval, reliable classification is even less likely during the less stable onset and offset transitions. However, the present findings do not establish performance during those transitions or over continuous operation. Future work should separately evaluate onset and offset dynamics and complete continuous-time operation in larger participant cohorts and intended end users.

## 6. Conclusions

This study investigated whether participant-specific four-finger classification performance after sensor re-donning could be recovered by adapting and reusing previously acquired Training data using a limited amount of labeled Calibration data from the current wearing condition. The proposed method transformed the prior Training signals toward the current condition through classwise amplitude correction and classwise covariance alignment, followed by feature re-extraction and classifier retraining.

The proposed Training-data adaptation recovered substantial re-donning-related degradation in Stable Motion classification. For P1, pooled accuracy across the ten Test datasets from five re-donning conditions increased from 88.90% with the fixed classifier to 98.88% after Classwise amplitude + Covariance adaptation. For P2 and P3, within exploratory post hoc failure-case subsets defined from the observed fixed-classifier degradation, accuracy increased from 24.46% to 98.97% and from 73.74% to 96.72%, respectively. Relative to classical feature-space CORAL references evaluated on the same splits, Global CORAL served only as an unsupervised reference under class-pooled covariance alignment, whereas Classwise CORAL provided the main classical comparison and remained at a level comparable to the proposed Combined adaptation. These results support the feasibility of class-specific amplitude and covariance adaptation for recovering finger discrimination when substantial cross-wearing distribution mismatch occurs within the evaluated participants and conditions. Amplitude correction provided the most consistent first-stage recovery in degraded conditions, whereas covariance and combined contributions varied across participants and wearing directions. These descriptive results establish participant-specific feasibility rather than a population-level ranking of methods.

The exploratory Calibration-repetition analysis further showed that Training-data reuse retained high classification accuracy when only a small number of current Calibration data were available. In this retrospective first-*r* subset analysis, with two Calibration repetitions per finger, Classwise amplitude + Covariance Training-data adaptation achieved 98.65%, 99.10%, and 95.92% for P1–P3, respectively. These values remained close to the 98.24%, 99.25%, and 97.38% obtained by Calibration-only classifiers trained using all five repetitions. Under the acquisition timing used in this study, reducing the Calibration protocol from five to two repetitions per finger would correspond to a theoretical reduction in recording duration from approximately 159 s to 87 s, or about 45%. Across the exhaustive retrospective shared-subset analyses, fold-nested two-repetition combined accuracy remained within 0.18–0.58 percentage points of the corresponding full-five accuracy for P1–P3.

Taken together, these findings support the feasibility of reusing previously acquired participant-specific Training data rather than reconstructing a classifier solely from newly acquired data after each sensor re-donning within the evaluated participants and wearing conditions. In particular, adapting prior Training data using a limited current Calibration set may reduce the amount of newly acquired labeled data required for personalized recalibration while maintaining high Stable Motion four-finger classification performance. Because the reduced-repetition conditions were evaluated retrospectively, two repetitions represent a promising setting for prospective validation rather than a confirmed minimum Calibration amount. The reported high Stable Motion accuracies therefore apply only to the standardized fixed-order isolated-motion protocol and should not be interpreted as evidence of order-independent finger classification or continuous practical operation. Overall, the results support the feasibility of reducing recalibration burden by adapting and reusing previously acquired participant-specific Training data after sensor re-donning.

## Figures and Tables

**Table 1:** Main comparison conditions used to evaluate the personalized recalibration framework.

Method	Transformation Target	Amplitude Correction	Covariance Alignment	Calibration Included in Training	SVM Retraining
Fixed classifier	None	None	None	No	No
Common8 + Fixed	Test data	Common 8-ch	None	No	No
Common8 retrain	Training data	Common 8-ch	None	No	Yes
Classwise amplitude	Training data	Class-specific 5 × 8	None	No	Yes
Classwise amplitude + Cal	Training data	Class-specific 5 × 8	None	Yes	Yes
Covariance	Training data	None	Classwise	No	Yes
Covariance + Cal	Training data	None	Classwise	Yes	Yes
Classwise amplitude + Covariance	Training data	Class-specific 5 × 8	Classwise	No	Yes
Classwise amplitude + Covariance + Cal	Training data	Class-specific 5 × 8	Classwise	Yes	Yes
Calibration only	None	None	None	Yes (only dataset)	Yes
Global CORAL	Training features	None	Global (feature space)	No	Yes
Classwise CORAL	Training features	None	Classwise (feature space)	No	Yes

**Table 2:** Descriptive data usage in the full five-repetition Calibration condition. Calibration quantities are mean ± SD across unique current-condition recordings (P1: 10; P2/P3: 6 each). The P1 Adapted Training value is the total number of eligible classifier-training windows in its single five-recording historical model. P2/P3 Adapted Training values are mean ± SD [range] across the three possible source-condition models, each constructed from two historical recordings. Covariance entries are raw 200-Hz samples; amplitude-reference and classifier-training entries are overlapping 300-ms windows updated every 60 ms. These counts describe algorithm-specific data use and are not equivalent numbers of independent observations.

Participant	Historical Recordings/Model	Adapted Training Windows/Model	Unique Cal Recordings	Cov Rest Raw Samples	Cov Motion Raw Samples	Amp-Ref Rest Windows	Amp-Ref Motion Windows	Cal-Only Training Windows	Cal-Only Unique Coverage [s]
P1	5	4370	10	19,187±308	12,535±307	1247±34	710±19	892±22	58.33±1.43
P2	2	1773±45[1726–1816]	6	19,289±254	12,432±260	1257±21	703±22	887±22	58.00±1.30
P3	2	1739±9[1732–1749]	6	19,486±234	12,229±225	1274±20	687±19	870±19	56.98±1.14

**Table 3:** Dataset-wise within-Training leave-one-dataset-out window-level accuracy for Participant 1.

Held-Out Training Dataset	Stable Windows	Accuracy	Misclassified Windows
Training 1	740	100.00%	0
Training 2	737	100.00%	0
Training 3	736	100.00%	0
Training 4	756	99.47%	4
Training 5	738	99.32%	5
Pooled	3707	99.76%	9

**Table 4:** Window-level accuracy and classwise recall of the fixed classifier for the ten held-out datasets of Participant 1 after sensor re-donning. Recall is reported separately for the four finger classes. No Calibration data were used in this comparison.

Re-Donning Condition	Held-Out Dataset	Stable Windows	Accuracy	Index Recall	Middle Recall	Ring Recall	Little Recall
Condition 1	Dataset 1	763	100.00%	100.00%	100.00%	100.00%	100.00%
Condition 1	Dataset 2	764	100.00%	100.00%	100.00%	100.00%	100.00%
Condition 2	Dataset 1	756	83.99%	92.51%	44.79%	100.00%	99.46%
Condition 2	Dataset 2	761	84.63%	92.27%	45.16%	100.00%	100.00%
Condition 3	Dataset 1	766	66.58%	52.02%	17.01%	100.00%	100.00%
Condition 3	Dataset 2	776	69.85%	77.16%	2.58%	100.00%	100.00%
Condition 4	Dataset 1	704	95.74%	95.29%	98.87%	100.00%	87.07%
Condition 4	Dataset 2	763	100.00%	100.00%	100.00%	100.00%	100.00%
Condition 5	Dataset 1	752	95.61%	86.91%	95.74%	100.00%	100.00%
Condition 5	Dataset 2	769	93.50%	94.92%	79.06%	100.00%	100.00%

**Table 5:** Window-level accuracy (%) of the compared recalibration methods for Participant 1. C1–C5 denote the five re-donning conditions, and each condition contains the pooled predictions from its two Calibration–Test directions. The highest pooled accuracy is shown in bold.

Method	C1	C2	C3	C4	C5	Pooled
Fixed classifier	100.00	84.31	68.22	97.96	94.54	88.90
Common8 + Fixed	100.00	94.73	89.30	97.96	98.42	96.05
Common8 retrain	100.00	94.33	87.61	97.96	98.36	95.62
Classwise amplitude	99.87	91.76	88.78	99.05	98.09	95.47
Classwise amplitude + Cal	99.80	96.04	96.04	99.25	99.34	98.09
Covariance	99.87	96.84	97.54	99.32	98.75	98.46
Covariance + Cal	99.93	96.64	98.18	99.39	98.82	98.59
Classwise amplitude + Covariance	99.87	97.82	98.25	99.32	99.15	**98.88**
Classwise amplitude + Covariance + Cal	99.87	97.17	98.18	99.18	99.08	98.69
Calibration only	99.02	97.89	96.82	99.32	98.22	98.24
Global CORAL	99.74	87.61	81.65	98.43	94.94	92.41
Classwise CORAL	98.82	98.48	96.43	99.32	97.30	98.06

**Table 6:** Participant 1 pooled window-level accuracy (%) according to the number of Calibration repetitions available for each finger class. Full Calibration used all confirmed repetitions available for each class. Reduced-repetition conditions were constructed retrospectively using the first *r* valid repetitions from recordings acquired with the full five-repetition protocol. The same 7574 held-out Stable Motion windows were used at every repetition count; only the Calibration subset available for model construction was varied. The highest accuracy in each row is shown in bold. Bold identifies the highest descriptive value and does not indicate superiority under matched classifier-training volume.

Calibration Repetitions	Calibration Only	Covariance- Adapted Training Data	Classwise Amplitude + Covariance-Adapted Training Data
1 repetition	93.90	97.36	**97.53**
2 repetitions	97.17	98.35	**98.65**
3 repetitions	98.06	**98.75**	98.57
Full Calibration	98.24	98.46	**98.88**

**Table 7:** Participant 1 classwise log-Euclidean covariance distances under full five-repetition Calibration.

No.	Comparison	Mean	SD	*n*
1	Training datasets (same wearing)	1.00	0.24	10
2	Calibration–Test (same wearing)	0.80	0.06	5
3	Training–Test (before adaptation)	1.46	0.26	10
4	Adapted training datasets	0.14	0.01	10
5	Adapted training–Calibration	0.16	0.02	10
6	Adapted training–Test	0.81	0.06	10

The value of *n* denotes the number of aggregation units. Condition 1 comprises the 10 pairwise comparisons among the five Training datasets. Condition 2 comprises one Calibration–Test comparison for each of the five re-donning conditions. Conditions 3, 5, and 6 comprise the 10 Calibration–Test folds. For Condition 4, pairwise distances among the five Adapted training datasets were averaged within each fold, yielding 10 fold-level values. Each value was first averaged over five classes, including Rest.

**Table 8:** Participant 3 window-level accuracy (%) across wearing conditions C1–C3. Diagonal entries show pooled reciprocal D1–D2 same-wearing validation. Off-diagonal entries show a fixed Training-data model constructed from both datasets in the row condition and evaluated on both datasets in the column condition without target-wearing Calibration.

Training Data	C1	C2	C3
C1	95.36	96.57	78.65
C2	95.02	95.00	84.09
C3	62.72	69.57	99.59

**Table 9:** Participant 2 directed cross-wearing window-level accuracy (%). Each value is the accuracy obtained with a model whose Training data combined D1 and D2 from the source condition, after pooling predictions from the two opposite assignments of D1 and D2 to the Calibration and Test roles within the target condition. P2 and P3 results were not pooled together. For P2, the four directions involving C1 were calibration-required; C2→C3 and C3→C2 were calibration-not-required.

Method	C1 Model		C2 Model		C3 Model	
	C1→C2	C1→C3	C2→C1	C2→C3	C3→C1	C3→C2
Fixed classifier	24.11	24.82	24.44	98.32	24.44	98.90
Common8 + Fixed	43.15	45.39	35.36	98.39	29.54	98.77
Common8 retrain	45.21	49.26	25.30	98.52	30.66	98.63
Classwise amplitude	94.59	96.13	96.56	98.26	99.01	98.49
Classwise amplitude + Cal	99.32	97.94	99.87	98.07	99.74	99.38
Covariance	98.84	98.97	99.01	98.32	97.95	98.84
Covariance + Cal	99.93	98.45	99.93	98.19	99.01	99.32
Classwise amplitude + Covariance	97.74	98.45	99.80	98.32	99.87	98.97
Classwise amplitude + Covariance + Cal	99.32	98.00	99.80	98.19	99.80	99.25
Calibration only	99.32	98.13	99.80	98.13	99.80	99.32
Global CORAL	46.99	40.68	44.24	99.10	40.20	98.63
Classwise CORAL	99.32	98.32	99.74	98.19	100.00	99.38

**Table 10:** Participant 3 directed cross-wearing window-level accuracy (%). Each value is the accuracy obtained with a model whose Training data combined D1 and D2 from the source condition, after pooling predictions from the two opposite assignments of D1 and D2 to the Calibration and Test roles within the target condition. P2 and P3 results were not pooled together. For P3, the four directions involving C3 were calibration-required; C1→C2 and C2→C1 were calibration-not-required.

Method	C1 Model		C2 Model		C3 Model	
	C1→C2	C1→C3	C2→C1	C2→C3	C3→C1	C3→C2
Fixed classifier	96.57	78.65	95.02	84.09	62.72	69.57
Common8 + Fixed	96.09	84.43	96.23	90.41	84.12	84.24
Common8 retrain	96.23	84.16	96.30	90.62	84.05	84.44
Classwise amplitude	95.75	98.57	96.43	97.28	92.73	91.36
Classwise amplitude + Cal	97.05	99.73	97.17	99.39	93.34	97.33
Covariance	95.07	99.46	95.22	98.03	90.85	91.84
Covariance + Cal	96.44	99.66	95.09	98.50	93.34	97.05
Classwise amplitude + Covariance	94.79	99.32	95.02	97.96	94.82	94.79
Classwise amplitude + Covariance + Cal	96.37	99.66	95.29	98.91	94.21	98.08
Calibration only	95.00	99.59	95.36	99.59	95.36	95.00
Global CORAL	95.96	76.82	94.21	79.40	72.41	73.82
Classwise CORAL	96.16	99.46	94.21	99.46	94.15	96.37

**Table 11:** Participant 2 window-level accuracy for the calibration-required pairs as a function of the number of target Calibration repetitions per finger. Reduced-repetition conditions were constructed retrospectively using the first *r* valid repetitions from recordings acquired with the full five-repetition protocol. The same 6031 held-out Stable Motion windows were used at every repetition count; only the Calibration subset available for model construction was varied. Bold identifies the highest descriptive value and does not indicate superiority under matched classifier-training volume.

Repetitions per Finger	Calibration Only (%)	Covariance (%)	Classwise Amplitude + Covariance (%)
1	95.09	97.50	**98.39**
2	98.14	98.26	**99.10**
3	98.69	98.84	**99.14**
5	**99.25**	98.69	98.97

**Table 12:** Participant 3 window-level accuracy for the calibration-required pairs according to the number of target Calibration repetitions per finger. Reduced-repetition conditions were constructed retrospectively using the first *r* valid repetitions from recordings acquired with the full five-repetition protocol. The same 5887 held-out Stable Motion windows were used at every repetition count; only the Calibration subset available for model construction was varied. Bold identifies the highest descriptive value and does not indicate superiority under matched classifier-training volume.

Repetitions per Finger	Calibration Only (%)	Covariance (%)	Classwise Amplitude + Covariance (%)
1	92.68	92.88	**95.01**
2	95.87	94.55	**95.92**
3	**96.82**	94.82	96.55
5	**97.38**	95.04	96.72

**Table 13:** Fold-nested Combined Stable Motion accuracy (%) for shared repetition-index subsets in the exhaustive supporting analysis. Values are mean ± SD across folds after within-fold subset averaging (P1: ten folds; P2/P3: 12 folds each, all six directed wearing-condition pairs). Subsets sharing repetitions were not treated as independent. $\Delta (r_{2}-r_{5})$ is the mean *r* = 2 accuracy minus the mean *r* = 5 accuracy. These summaries differ from the window-pooled first-*r* primary Calibration-amount tables.

Participant	*r* = 1	*r* = 2	*r* = 3	*r* = 5	$\Delta (r_{2}-r_{5})$ [pp]
P1	97.75±1.50	98.29±1.42	98.54±1.22	98.87±0.93	−0.58
P2	98.09±1.38	98.68±1.13	98.79±1.16	98.86±1.22	−0.18
P3	94.36±4.63	95.58±4.15	95.85±4.18	96.04±4.04	−0.47

**Table 14:** Adaptation-parameter stability across shared repetition-index subsets (Combined pipeline). Values are mean ± SD of fold-level subset means. Log-gain difference is the Frobenius norm between subset and full-five log-gain matrices; covariance difference is the mean classwise log-Euclidean distance between subset and full-five Calibration covariance estimates (*r* = 5: zero by definition). One unavailable P1 single-repetition subset was omitted before that fold’s *r* = 1 mean.

Participant	*r*	Log-Gain Difference	Covariance Difference
P1	1	0.539±0.044	0.402±0.025
	2	0.334±0.028	0.252±0.025
	3	0.221±0.024	0.168±0.017
P2	1	0.615±0.082	0.434±0.036
	2	0.380±0.038	0.264±0.021
	3	0.258±0.039	0.176±0.014
P3	1	0.778±0.030	0.515±0.021
	2	0.505±0.054	0.316±0.017
	3	0.333±0.026	0.210±0.012

**Table 15:** Paired accuracy differences (percentage points) between recalibration methods on the same evaluation units. P1 uses the ten held-out Test datasets; P2 and P3 use the six directed wearing-condition pairs. Amplitude–Fixed is the first-stage amplitude contribution. Combined–Amplitude is the sequential increment obtained by adding classwise covariance alignment after amplitude correction. Combined–Covariance compares the proposed Combined pipeline with covariance-only adaptation. Positive/zero/negative counts refer to the sign of each paired difference. These summaries are descriptive within-participant comparisons and are not population-level significance tests.

Participant	Comparison	Unit	*n*	Pos	Zero	Neg	Mean Δ
P1	Amplitude–Fixed	Test dataset	10	7	1	2	+6.51
P1	Combined–Amplitude	Test dataset	10	8	2	0	+3.37
P1	Combined–Covariance	Test dataset	10	6	4	0	+0.42
P2	Amplitude–Fixed	Direction	6	4	0	2	+48.00
P2	Combined–Amplitude	Direction	6	6	0	0	+1.69
P2	Combined–Covariance	Direction	6	3	1	2	+0.21
P3	Amplitude–Fixed	Direction	6	5	0	1	+14.25
P3	Combined–Amplitude	Direction	6	4	0	2	+0.76
P3	Combined–Covariance	Direction	6	2	0	4	+1.04

**Table 16:** Evaluation-unit Stable Motion accuracy summaries for the main recalibration methods, reported to make cross-dataset and cross-direction variability explicit alongside the window-pooled accuracies. Units are the ten held-out Test datasets for P1 and the six directed wearing-condition pairs for P2/P3. Values are unweighted across units and therefore need not equal the corresponding window-pooled accuracies. IQR denotes the interquartile range. The 95% interval is an evaluation-unit bootstrap interval for the mean unit-level accuracy (B=10,000), resampling evaluation units rather than overlapping windows. Directed pairs for P2/P3 reuse recordings across source/target roles, so the intervals are descriptive within-participant summaries rather than population-level inferential intervals.

Participant	Unit	Method	*n*	Mean ± SD	Median	IQR	95% EU Boot	Range
P1	Test dataset	Fixed	10	88.99±12.41	94.55	[83.99,100]	[81.32,95.57]	[66.58,100]
P1	Test dataset	Amplitude	10	95.50±5.00	97.95	[90.21,99.74]	[92.43,98.20]	[87.37,100]
P1	Test dataset	Covariance	10	98.45±1.31	98.56	[97.09,99.74]	[97.69,99.21]	[96.58,100]
P1	Test dataset	Combined	10	98.87±0.93	98.95	[98.02,99.74]	[98.34,99.40]	[97.52,100]
P2	Direction	Fixed	6	49.17±38.30	24.63	[24.44,98.32]	[24.39,74.05]	[24.11,98.90]
P2	Direction	Amplitude	6	97.17±1.70	97.41	[96.13,98.49]	[95.90,98.34]	[94.59,99.01]
P2	Direction	Covariance	6	98.65±0.42	98.84	[98.32,98.97]	[98.31,98.93]	[97.95,99.01]
P2	Direction	Combined	6	98.86±0.85	98.71	[98.32,99.80]	[98.26,99.46]	[97.74,99.87]
P3	Direction	Fixed	6	81.10±13.56	81.37	[69.57,95.02]	[71.46,90.73]	[62.72,96.57]
P3	Direction	Amplitude	6	95.36±2.76	96.09	[92.73,97.28]	[93.28,97.20]	[91.36,98.57]
P3	Direction	Covariance	6	95.08±3.35	95.14	[91.84,98.03]	[92.61,97.46]	[90.85,99.46]
P3	Direction	Combined	6	96.12±2.00	94.92	[94.79,97.96]	[94.83,97.62]	[94.79,99.32]

**Table 17:** Evaluation-unit bootstrap summaries of the paired method differences in Table 15. Mean ± SD, median, IQR, and the 95% evaluation-unit bootstrap interval are computed on the same evaluation-unit differences (percentage points). Intervals that include zero indicate that the mean paired difference was not stably positive or negative under unit-level resampling. As for Table 16, these intervals are descriptive within-participant summaries rather than population-level inferential intervals.

Participant	Comparison	Unit	*n*	Mean ± SD	Median	IQR	95% EU Boot
P1	Amplitude–Fixed	Test dataset	10	+6.51±8.23	+3.39	[0.00,10.64]	[2.23,11.76]
P1	Combined–Amplitude	Test dataset	10	+3.37±4.45	+1.06	[0.26,7.31]	[1.01,6.11]
P1	Combined–Covariance	Test dataset	10	+0.42±0.42	+0.39	[0.00,0.91]	[0.18,0.67]
P2	Amplitude–Fixed	Direction	6	+48.00±37.39	+70.89	[−0.06,72.12]	[23.42,72.53]
P2	Combined–Amplitude	Direction	6	+1.69±1.40	+1.59	[0.48,3.15]	[0.67,2.68]
P2	Combined–Covariance	Direction	6	+0.21±1.05	+0.07	[−0.52,0.79]	[−0.50,1.01]
P3	Amplitude–Fixed	Direction	6	+14.25±12.09	+16.55	[1.41,21.80]	[5.65,22.79]
P3	Combined–Amplitude	Direction	6	+0.76±1.82	+0.71	[−0.96,2.09]	[−0.54,2.08]
P3	Combined–Covariance	Direction	6	+1.04±1.90	−0.10	[−0.20,2.95]	[−0.18,2.43]

**Table 18:** Complementary pooled Stable Motion metrics for the main methods. Accuracy is the primary window-pooled endpoint used throughout the Results. Balanced accuracy is the unweighted mean of Index, Middle, Ring, and Little recall, and macro-F1 is the unweighted mean of the four classwise F1-scores. For P1, values pool all ten held-out Test datasets. For P2 and P3, values pool the exploratory post hoc calibration-required failure-case directions.

Participant	Scope	Method	Accuracy	Balanced Acc.	Macro-F1
P1	Ten Test datasets	Fixed	88.90	88.93	87.77
P1	Ten Test datasets	Amplitude	95.47	95.50	95.27
P1	Ten Test datasets	Covariance	98.46	98.46	98.44
P1	Ten Test datasets	Combined	98.88	98.88	98.87
P1	Ten Test datasets	Global CORAL	92.41	92.32	92.22
P1	Ten Test datasets	Classwise CORAL	98.06	98.04	98.04
P2	Cal.-required pool	Fixed	24.46	25.00	9.83
P2	Cal.-required pool	Amplitude	96.58	96.61	96.57
P2	Cal.-required pool	Covariance	98.69	98.68	98.69
P2	Cal.-required pool	Combined	98.97	98.98	98.98
P2	Cal.-required pool	Global CORAL	42.98	42.87	43.21
P2	Cal.-required pool	Classwise CORAL	99.34	99.34	99.34
P3	Cal.-required pool	Fixed	73.74	73.56	73.82
P3	Cal.-required pool	Amplitude	94.99	94.94	94.94
P3	Cal.-required pool	Covariance	95.04	94.99	95.06
P3	Cal.-required pool	Combined	96.72	96.68	96.69
P3	Cal.-required pool	Global CORAL	75.61	75.54	75.60
P3	Cal.-required pool	Classwise CORAL	97.35	97.33	97.32

**Table 19:** Condition- and direction-level descriptive contributions along the proposed recalibration sequence. ΔAmp = Amplitude–Fixed and Δ(Comb–Amp) = Combined–Amplitude (percentage points), so the latter is the sequential increment after amplitude correction rather than a comparison between independent Covariance-only and Amplitude-only methods. For P1, each row is the window-pooled accuracy of the two held-out Test datasets within the corresponding re-donning condition. Physical electrode displacement was not measured and is not inferred here.

Participant	Unit	Fixed	ΔAmp	Δ(Comb–Amp)
P1	C1	100.00	−0.13	0.00
P1	C2	84.31	+7.45	+6.06
P1	C3	68.22	+20.56	+9.47
P1	C4	97.96	+1.09	+0.27
P1	C5	94.54	+3.55	+1.06
P2	C1→C2	24.11	+70.48	+3.15
P2	C1→C3	24.82	+71.31	+2.32
P2	C2→C1	24.44	+72.12	+3.25
P2	C2→C3	98.32	−0.06	+0.06
P2	C3→C1	24.44	+74.57	+0.86
P2	C3→C2	98.90	−0.41	+0.48
P3	C1→C2	96.57	−0.82	−0.96
P3	C1→C3	78.65	+19.92	+0.75
P3	C2→C1	95.02	+1.41	−1.41
P3	C2→C3	84.09	+13.19	+0.68
P3	C3→C1	62.72	+30.01	+2.09
P3	C3→C2	69.57	+21.80	+3.43

**Table 20:** Window-level accuracy (%) of the fixed classifier with class-independent Test-side correction for Participant 1. C1–C5 denote the five re-donning conditions, and each condition contains the pooled predictions from its two Calibration–Test directions. No classifier retraining was performed. GlobalCov denotes pooled Motion covariance alignment. GlobalCorr denotes pooled Motion correlation alignment applied after Common8 while retaining the Common8-corrected channel variances. Fixed indicates that the original classifier was used without retraining.

Method	C1	C2	C3	C4	C5	Pooled
Fixed classifier	100.00	84.31	68.22	97.96	94.54	88.90
Common8 + Fixed	100.00	**94.73**	**89.30**	97.96	**98.42**	**96.05**
GlobalCov + Fixed	100.00	89.26	83.79	98.23	97.44	93.69
Common8 + GlobalCorr + Fixed	99.87	88.40	83.01	**98.43**	96.12	93.11

## Data Availability

The core offline MATLAB code implementing classwise amplitude correction, covariance alignment, log-covariance feature extraction, classifier retraining, and evaluation is available from the corresponding author upon reasonable request. De-identified, non-reconstructive derived feature data and aggregate analysis outputs that do not contain raw sEMG time-series samples can also be provided upon reasonable request, subject to applicable institutional and ethical requirements. Raw participant recordings and trained participant-specific models are not publicly available because the original consent and ethics framework did not authorize unrestricted public distribution of participant biosignals, and these materials may retain participant-specific information even after removal of direct identifiers. Device-control software is not publicly available because it is system-specific.

## Abbreviations

The following abbreviations are used in this manuscript:

ECOC	Error-correcting output codes
HMI	Human–machine interface
RMS	Root mean square
sEMG	Surface electromyography
SVM	Support vector machine
